# Multidimensional neuropsychological heterogeneity in obstructive sleep apnea: deep clinical phenotyping in a single-center cohort, population proteomic context, and depressive-burden risk stratification

**DOI:** 10.3389/fimmu.2026.1874192

**Published:** 2026-09-15

**Authors:** Xiaoyu Wang, Yirun Jiang, Leilei Yu, Pengfei Ye, Chao Li, Chutian Xing, Wen Hu, Zhi Qiao, Xianshan Hu, Shizhen Zou, Siyuan Hou, Rui Zhang, Jinrang Li, Jun Tai

**Affiliations:** 1 Capital Institute of Pediatrics, Chinese Academy of Medical Sciences & Peking Union Medical College, Beijing, China; 2 Capital Center for Children’s Health, Capital Medical University, Capital Institute of Pediatrics, Beijing, China; 3 College of Otolaryngology Head and Neck Surgery, The 6th Medical Center, National Clinical Research Center for Otolaryngologic Diseases, Chinese PLA General Hospital, Beijing, China

**Keywords:** cognition, depression, immunometabolic stress, machine learning, obstructive sleep apnea, population proteomics, sleep fragmentation, TyG-WHtR

## Abstract

Obstructive sleep apnea (OSA) is associated with substantial cognitive and affective burden, yet conventional respiratory severity does not fully explain this heterogeneity. We investigated whether neuropsychological vulnerability in OSA shows differentiated clinical association patterns, whether cognition, depression, and anxiety share general-population proteomic associations, and whether a separate depressive-burden model can support pragmatic risk stratification. Methods: We conducted a clinically anchored study integrating a single-center clinically phenotyped adult OSA cohort, plasma proteomics in the UK Biobank Olink subset, machine-learning-based candidate prioritization, a translational extension based on TyG-WHtR-anchored depressive-burden stratification, and longitudinal human PBMC transcriptomic contextualization. In the single-center cohort, multivariable regression and exploratory cross-sectional decomposition were used to evaluate associations among respiratory burden, sleep fragmentation, metabolic dysregulation, cognition, and affective symptoms. In the UK Biobank proteomic dataset, proteins associated with cognition, depression, and anxiety were identified after full-proteome false discovery rate correction. The risk model was developed in available-source OSA and clinically evaluated, without refitting, in a single-center polysomnography-defined OSA cohort. Results: In the clinically phenotyped cohort, cognition was associated with both apnea burden and TyG, whereas affective burden was more strongly associated with TyG; the cross-sectional decompositions do not establish causal mediation. Of 2,922 estimable proteins per outcome, 886, 152, and 45 were FDR-significant for cognition, PHQ-2, and GAD-2, respectively, with 21 shared across all three outcomes and showing non-random adverse-direction concordance. Strict mutual-outcome and CRP/SII adjustment attenuated cross-outcome significance; in OSA-context analyses, directional concordance remained common, although individual associations did not survive FDR correction. Of 117 machine-learning workflows, 103 were evaluable; the formally selected workflow achieved locked-validation AUROC 0.694. The primary full-spectrum risk model achieved nested out-of-fold AUROC 0.726 and clinical-evaluation AUROC 0.809; calibration was less stable across settings (Brier score 0.356), indicating that absolute probabilities may require local recalibration. In the PBMC analysis, pathway-level changes involved immune, lipid/lysosomal, mitochondrial/redox, and vascular/barrier processes; predefined candidate-gene testing was not FDR-significant. Conclusion: The findings support a multidimensional framework integrating differentiated clinical associations, a non-random general-population proteomic context, human functional transcriptomic context, and encouraging cross-setting depressive-burden risk ranking, while absolute probabilities remained less stable; OSA-specific molecular validation and prospective calibration remain priorities.

## 1 Introduction

Obstructive sleep apnea (OSA) is a common sleep-related breathing disorder characterized by recurrent upper airway collapse, intermittent hypoxia, fluctuations in intrathoracic pressure, and frequent awakenings during sleep (1–3). The clinical impact of OSA is not limited to daytime sleepiness. A growing body of research indicates that OSA is closely associated with cognitive decline, depressive symptoms, and anxiety (4–10). Previous studies have shown that this neuropsychological impairment is not uniform across patients. Clinically, it is common to observe patients with similar degrees of respiratory obstruction exhibiting markedly different cognitive and emotional manifestations, suggesting that traditional measures such as the apnea-hypopnea index cannot fully account for this heterogeneity in neuropsychological impairment (11–14).

This heterogeneity may reflect multiple interrelated physiological stressors. Intermittent hypoxia is a central component of OSA pathophysiology (15–17), whereas recurrent arousals and sleep fragmentation represent another core consequence of repeated obstructive events (1, 2). Experimental and clinical literature links these recurrent stressors with neurocognitive and systemic oxidative, inflammatory, and vascular consequences (11–20). OSA is increasingly recognized as a systemic disease characterized by immune activation, endothelial dysfunction, and immunometabolic stress (18–20). These observations therefore motivated us to examine whether cognition and mood show different associations with respiratory, sleep-fragmentation, metabolic, and circulating molecular features.

Recent advances in biological technologies and artificial intelligence have expanded biomedical research capabilities. These advances include proteomic assays that profile large numbers of circulating proteins and machine-learning methods that analyze high-dimensional data and prioritize candidate features. Transcriptomic analyses may provide additional molecular context for clinical phenomena. However, how respiratory burden, sleep fragmentation, metabolic features, and circulating molecular correlates relate to different dimensions of neuropsychological burden remains incompletely characterized. Furthermore, it remains unclear whether sleep fragmentation and metabolic dysregulation show consistent associations with neuropsychological vulnerability in OSA, or whether accessible metabolic measures can assist depressive-burden risk stratification.

We conducted a multi-layered translational study to investigate the clinical and molecular context of neuropsychological vulnerability in OSA. Using an OSA cohort from the Department of Laryngology and Voice Surgery at the Sixth Medical Center of the Chinese PLA General Hospital, we investigated whether respiratory burden, sleep fragmentation, and metabolic measures showed differentiated cross-sectional associations with cognitive and affective outcomes. We then leveraged UK Biobank Olink plasma proteomics to determine whether cognitive, depressive, and anxiety outcomes show overlapping protein associations in the general population. Separately, we applied 117 predefined machine-learning workflows for exploratory proteomic feature prioritization and used human PBMC transcriptomic data to provide additional functional context for predefined candidate signals. Finally, we explored whether TyG-WHtR, as a clinically accessible metabolic measure, could contribute to depressive-burden risk ranking and evaluated the locked model in a PSG-defined single-center clinical cohort (**Figure 1**).

**Figure 1:**
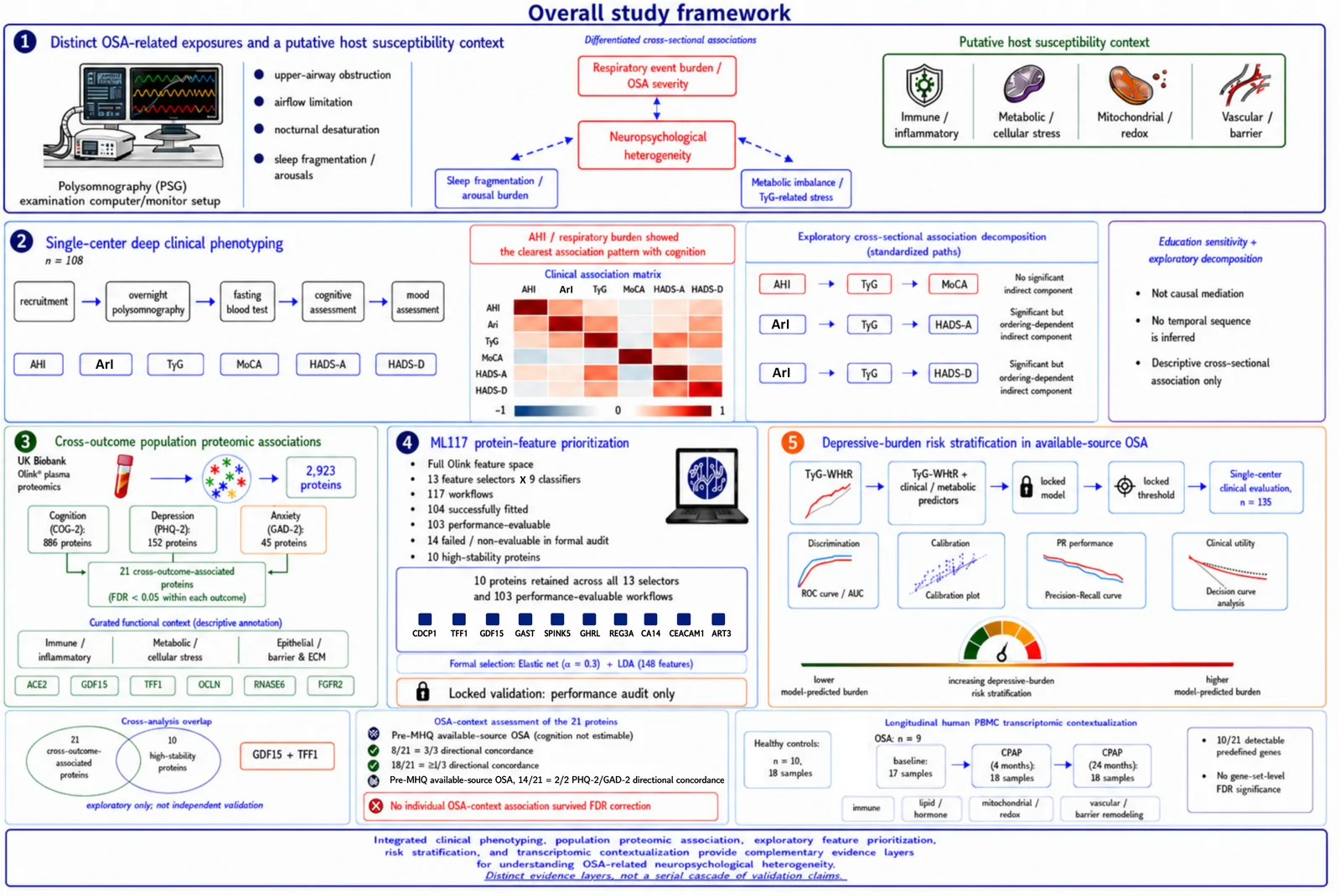
Overall study framework integrating clinical phenotyping, population proteomics, machine-learning prioritization, depressive-burden risk stratification, and transcriptomic contextualization in obstructive sleep apnea. The framework includes deep phenotyping in a single-center PSG-defined OSA cohort (n, 108); full-proteome analysis of 2,923 Olink proteins for cognition, PHQ-2, and GAD-2; 117 prespecified machine-learning workflows for protein-feature prioritization; a depressive-burden risk model developed using nested out-of-fold estimation and clinically evaluated in 135 single-center participants; OSA-context analyses of the 21 cross-outcome proteins; and longitudinal human PBMC transcriptomic contextualization. The components represent distinct evidence layers and do not constitute a serial causal or validation chain.

## 2 Materials and methods

### 2.1 Study design

This study was designed as a translational investigation integrating deep clinical phenotyping, peripheral proteomics, candidate prioritization, transcriptomic contextualization, and clinical extension. The overall design integrated clinical phenotypes, peripheral proteomics, machine-learning-based selection, and clinical risk stratification within a single research framework (**Figure 1**). The study comprised five analytically distinct components. The first component was a deep clinical phenotyping cohort of adults with OSA recruited at the Department of Laryngology and Voice Surgery, Sixth Medical Center of the Chinese PLA General Hospital, used for baseline characterization of cognitive, emotional, and metabolic features. The second component involved general-population UK Biobank Olink plasma proteomics analysis to identify proteins associated with cognition, depression, and anxiety. The third component included prespecified machine-learning workflows for candidate-protein prioritization. The fourth component was a translational extension module that developed a depressive-burden risk model in available-source UK Biobank OSA and clinically evaluated the locked model in a single-center PSG-defined OSA dataset. The fifth component used a public longitudinal human PBMC RNA-sequencing dataset for OSA/CPAP transcriptomic contextualization. The cohorts, outcome instruments, and analytical roles differed and were not treated as interchangeable or as independent biological replication (**Supplementary Table 13**).

Human studies were approved by the Ethics Committee of the Sixth Medical Center of the Chinese PLA General Hospital (approval number HZKY-PJ-2025-7). All participants in the cohort from the Sixth Medical Center of the Chinese PLA General Hospital signed written informed consent forms. Analysis of UK Biobank data was conducted under approved application number 575800. The public human PBMC transcriptomic dataset was analyzed in accordance with the usage guidelines of the database.

### 2.2 Deep phenotyping OSA cohort at the Sixth Medical Center of the Chinese PLA General Hospital

The OSA cohort was a cross-sectional clinical study conducted from September 27, 2025, to February 6, 2026, and enrolled adults with OSA who visited the Department of Laryngology and Voice Surgery at the Sixth Medical Center of the Chinese PLA General Hospital. Inclusion criteria included being at least 18 years of age, completing an interpretable overnight PSG, undergoing a single morning fasting biochemical test, and participating in a standardized neuropsychological assessment. Exclusion criteria included a history of OSA-specific treatment, the presence of major neurological disorders, severe systemic diseases, active psychiatric disorders likely to significantly affect neuropsychological assessment, pregnancy or lactation, and missing key data.

Daytime sleepiness was assessed before PSG using the Epworth Sleepiness Scale (ESS) under the guidance of trained staff. The ESS has established internal consistency across studies (24), and prior work from the same center also used PSG and ESS in a large OSAHS cohort (64). The eight standard situations were each scored from 0 (no chance of dozing) to 3 (high chance of dozing), yielding a total score of 0–24; in the present study, an ESS score ≥9 was prespecified to classify daytime sleepiness.

For overnight PSG, participants were asked to arrive at the sleep center at least 2 h before their habitual bedtime. On the monitoring day, alcohol, coffee or other caffeine-containing beverages, sedative-hypnotic medications, and other sleep-affecting agents were prohibited. PSG was performed in a quiet, darkened sleep laboratory with appropriate temperature and humidity using a multifunctional sleep-monitoring system. Recorded signals included six electroencephalographic derivations (F4-M1, C4-M1, O2-M1, F3-M2, C3-M2, and O1-M2), electrocardiography, bilateral electrooculography, oronasal airflow, pulse oxygen saturation (SaO2) recorded from an index finger, thoracoabdominal respiratory movements, submental/chin electromyography, bilateral leg electromyography, and body position. A complete overnight recording with at least 4 h of valid monitoring was required.

All PSG recordings were manually scored by trained sleep technologists, according to established AASM criteria (21). OSA was diagnosed based on an apnea-hypopnea index (AHI) ≥5 events/h and was classified as mild (5 ≤ AHI < 15 events/h), moderate (15 ≤ AHI < 30 events/h), or severe (AHI ≥30 events/h). For the lowest oxygen saturation (LSaO2), values of 85% ≤ LSaO2 < 90%, 65% ≤ LSaO2 < 85%, and LSaO2 < 65% were categorized as mild, moderate, and severe hypoxemia, respectively. Primary PSG parameters included AHI, arousal index (ArI), oxygen desaturation index, mean oxygen saturation, minimum oxygen saturation, total sleep time, sleep efficiency, and the proportion of time spent in each sleep stage.

Fasting blood glucose, triglycerides, and high-density lipoprotein cholesterol were measured the morning after PSG. The triglyceride-glucose index (TyG) was calculated using the natural logarithm as TyG, ln{[fasting triglycerides (mg/dL) × fasting plasma glucose (mg/dL)]/2}, consistent with the original TyG formulation and subsequent clamp-validation work (65, 66). Neuropsychological and questionnaire assessments were completed before PSG during the same clinical visit. Cognitive performance was assessed face-to-face using the Chinese-language Montreal Cognitive Assessment, Beijing version (MoCA-Beijing), administered by a professionally trained sleep-medicine physician (22). The MoCA-Beijing yields a total score of 0–30; 1 point was added for participants with 12 or fewer years of formal education, with the corrected score capped at 30. MoCA was analyzed as a continuous measure of cognitive performance rather than to classify cognitive impairment by a fixed cutoff. Chinese-language versions of the Hospital Anxiety and Depression Scale (HADS) and ESS were administered under standardized instructions. HADS-A and HADS-D were analyzed as continuous symptom-burden measures; HADS-D screening thresholds, where used descriptively, were not treated as psychiatric diagnoses (23).

### 2.3 Clinical correlations and cross-sectional decomposition analysis

First, we conducted correlation analyses of demographic, sleep-related, metabolic, cognitive, and emotional variables in the OSA cohort to describe the overall clinical profile. Subsequently, multivariable linear regression models were constructed to assess the independent associations between AHI, ArI, and TyG and education-corrected MoCA, HADS-A, and HADS-D after adjusting for age, sex, and body mass index (BMI). AHI, ArI, and TyG were included simultaneously in these models to distinguish the relative contributions of respiratory burden, sleep fragmentation, and metabolic imbalance to different neuropsychological dimensions; the MoCA model additionally included continuous years of education. HC3 heteroscedasticity-consistent standard errors were used, and Benjamini-Hochberg correction was applied across the nine primary predictor-outcome tests (**Figures 2A, B**; **Supplementary Table 1).**

**Figure 2:**
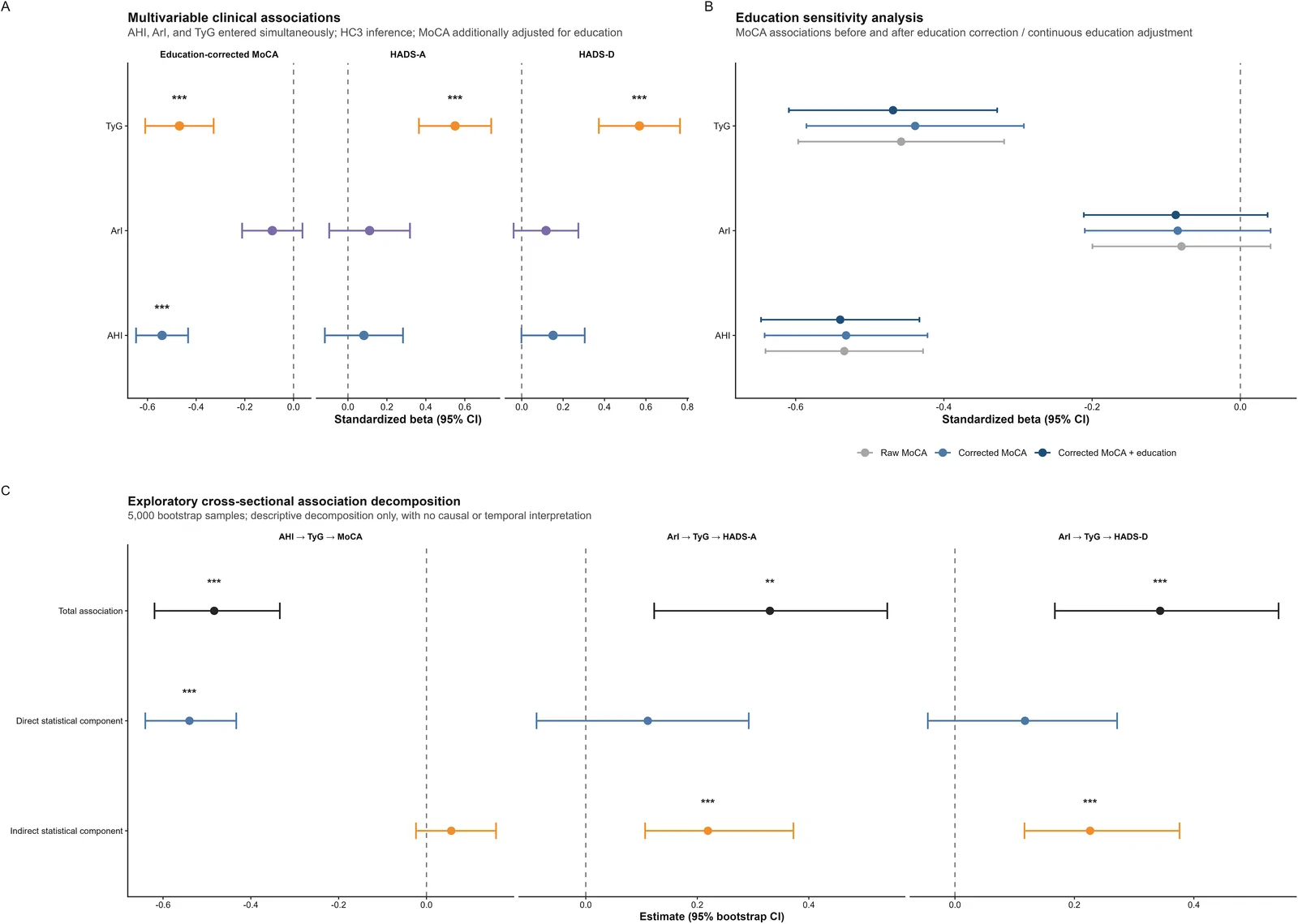
Clinical associations and sensitivity analyses in the single-center cohort. **(A)** Multivariable associations of AHI, ArI, and TyG with MoCA, HADS-A, and HADS-D, adjusted for age, sex, and BMI; MoCA models were additionally adjusted for education. Standardized beta coefficients with 95% CIs are shown. **(B)** Sensitivity analyses of MoCA associations under different education-adjustment strategies. **(C)** Exploratory decomposition of AHI -> TyG -> MoCA, ArI -> TyG -> HADS-A, and ArI -> TyG -> HADS-D into total, direct, and indirect statistical components using 5,000 bootstrap resamples. These cross-sectional analyses are descriptive and do not imply causal mediation. Competing-exposure mutual-adjustment sensitivity analyses are reported in **Supplementary Table 1B**. AHI, apnea-hypopnea index; ArI, arousal index; TyG, triglyceride-glucose index; MoCA, Montreal Cognitive Assessment; HADS-A/D, Hospital Anxiety and Depression Scale-Anxiety/Depression; BMI, body mass index; CI, confidence interval.

We predefined three cross-sectional orderings to describe whether metabolic imbalance statistically accounted for part of the association between sleep-related exposures and neuropsychological outcomes. These orderings were: AHI -> TyG -> education-corrected MoCA, ArI -> TyG -> HADS-A, and ArI -> TyG -> HADS-D. For each ordering, we calculated indirect, direct, and total statistical components using 5,000 nonparametric bootstrap samples, adjusting for age, sex, and BMI (**Figure 2C**; **Supplementary Table 1**). Reverse-order decompositions were also examined. As a competing-exposure sensitivity analysis, ArI was additionally included in both component regressions for the AHI -> TyG -> education-corrected MoCA ordering, whereas AHI was additionally included in both component regressions for the ArI -> TyG -> HADS-A and ArI -> TyG -> HADS-D orderings. The age, sex, and BMI covariate specification was otherwise unchanged, and MoCA models retained continuous education adjustment. Corresponding exposure-outcome and exposure-TyG associations were estimated under the same specifications (**Supplementary Table 1B**). Because all variables were measured cross-sectionally, no temporal sequence or causal mediation is inferred.

### 2.4 UK biobank Olink proteomics analysis

Participants from the UK Biobank who had Olink plasma proteomics data, as well as relevant covariates and questionnaire information, were included (25, 26). Plasma proteins were quantified using Olink proximity extension assay technology, whose analytical principles and performance have been described previously (27–29). Protein abundance was represented by normalized protein expression (NPX), a relative log2-scale expression measure. The proteomics data were matched with clinical and questionnaire information based on participant IDs for subsequent analysis. This proteomic analysis used the UK Biobank general-population Olink subset and did not require an OSA diagnosis.

This study defined three outcomes to assess neuropsychological vulnerability at the population level. The cognitive composite score, COG_z, was constructed from the baseline assessment (instance 0) reaction-time measure (UK Biobank Data-Field 20023; p20023_i0) and fluid-intelligence measure (Data-Field 20016; p20016_i0). Reaction time was standardized and sign-reversed, while fluid intelligence was standardized directly; the two standardized measures were then averaged, with both components required for the primary composite, such that lower COG_z values indicate poorer cognitive performance. Depressive and anxiety symptom outcomes were reconstructed from the same online Mental Health Questionnaire (MHQ) wave: PHQ-2 from Data-Fields 20510 and 20514, and GAD-2 from Data-Fields 20506 and 20509. Item responses were scored on 0-to-3 scales and summed to 0-6; a total score of 3 or higher defined probable depressive- and anxiety-symptom endpoints, respectively. Thus, cognition was based on the baseline assessment, whereas PHQ-2 and GAD-2 were derived from the same later MHQ wave; the two affective outcomes were measured in the same wave, not with the baseline cognitive assessment.

For each of 2,923 QC-analyzable proteins, three separate regression models were constructed, adjusted for age, sex, and BMI; 2,922 proteins were estimable per outcome. COG_z was analyzed using multivariable linear regression, while PHQ-2 depression and GAD-2 anxiety were analyzed using multivariable logistic regression. Protein NPX was standardized to 1 SD, and P-values for each outcome were adjusted for FDR using the Benjamini-Hochberg method across the full tested proteome. Proteins that met an FDR of less than 0.05 across all three outcomes were defined as cross-outcome associated proteins and were further classified by effect direction (**Figure 3**; **Supplementary Table 2**). Pearson correlations among COG_z, PHQ-2, and GAD-2 were estimated in the strict-adjustment common complete-case sample (n, 5,296; complete data for the three outcomes, CRP, and SII). Directional concordance was further examined using 5,000 joint permutations that applied the same participant-level permutation index to all three outcomes within age-by-sex strata, 10,000 missingness-matched random protein sets, and strict models additionally adjusting for the other two outcomes, CRP, and SII (**Supplementary Table 3**). No additional *post-hoc* effect-size threshold was imposed because no prespecified or established cross-outcome minimum effect criterion was available; effect magnitude was therefore reported alongside FDR rather than used for retrospective selection.

**Figure 3:**
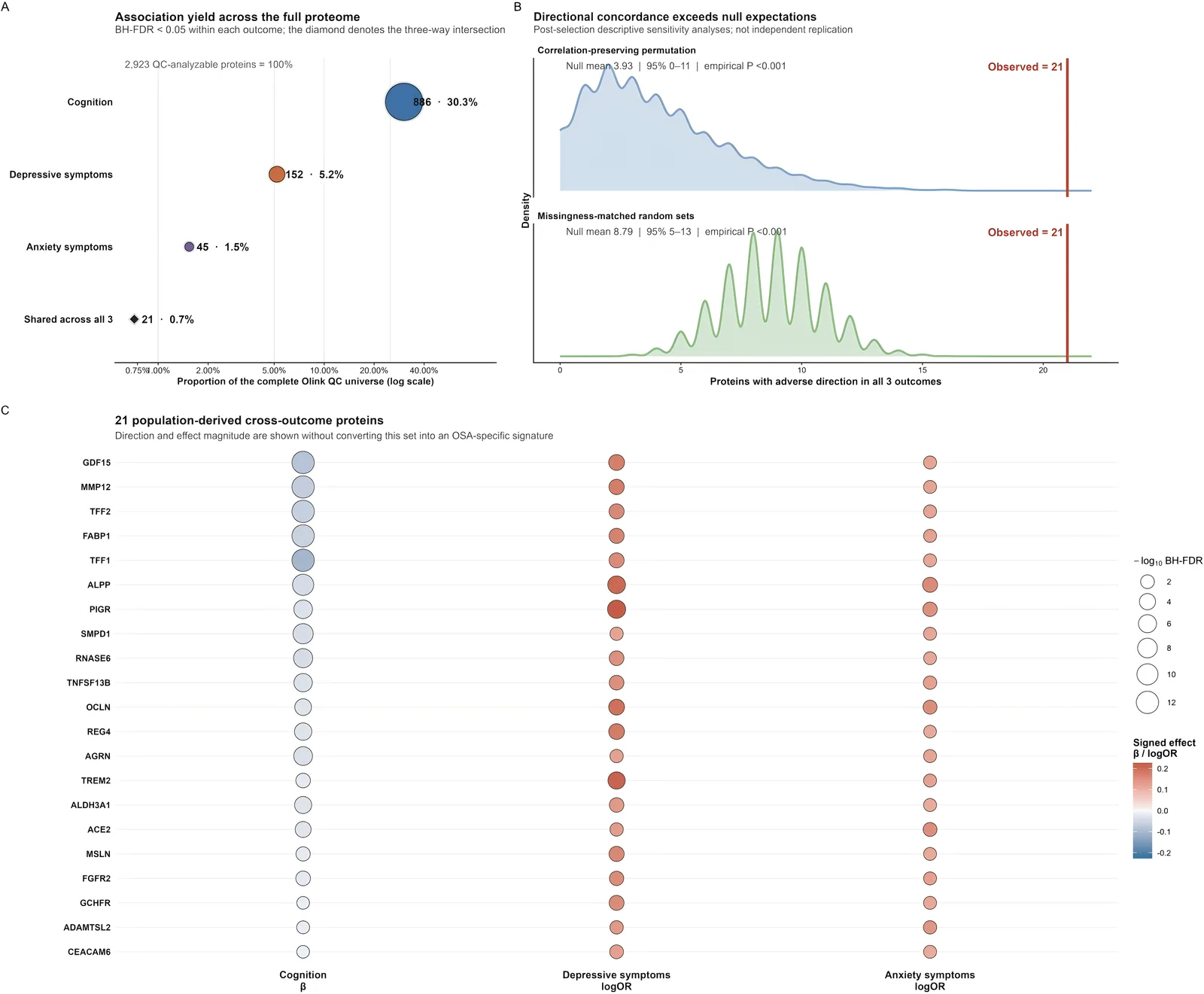
Full-proteome population analysis identifies a 21-protein cross-outcome association set and directional concordance beyond null expectations. **(A)** Among 2,923 QC-analyzable proteins, 886, 152, and 45 met BH-FDR < 0.05 for cognition, PHQ-2, and GAD-2, respectively; 21 were significant across all three outcomes. **(B)** Correlation-preserving joint permutation and missingness-matched random-set analyses compared the observed 21/21 adverse-direction concordance with post-selection null expectations. **(C)** In the cross-outcome bubble matrix, color represents standardized beta or log OR and area represents -log10(BH-FDR), truncated at 12. Proteins are ordered by summed -log10(BH-FDR). Models were adjusted for age, sex, and BMI, and BH correction was applied over the full tested proteome for each outcome. BH-FDR, Benjamini-Hochberg false discovery rate; BMI, body mass index; log OR, log odds ratio; NPX, normalized protein expression.

For OSA-context analyses, OSA was reconstructed at the participant level from the source fields used in the finalized analysis. Available-source OSA was defined by self-reported sleep apnea in UK Biobank Data-Field 20002 (code 1123) and/or hospital inpatient ICD-10 sleep apnea in Data-Field 41270 (G47.3). Repeated self-report arrays and repeated inpatient records were collapsed to participant-level indicators. Hospital diagnosis dates were obtained from the corresponding Data-Field 41280 date arrays; when ICD-10 codes were stored as collapsed tokens, token positions were paired with the matching 41280 array indices. MHQ completion date was taken from Data-Field 20400. The primary pre-MHQ available-source definition required either baseline (instance 0) self-reported sleep apnea or an inpatient G47.3 diagnosis dated on or before MHQ completion. The ever-recorded available-source definition accepted any qualifying self-report or inpatient G47.3 record without temporal restriction. Pre-MHQ ICD-defined OSA required an inpatient G47.3 diagnosis dated on or before MHQ completion, whereas ever-recorded ICD-defined OSA accepted any inpatient G47.3 record irrespective of timing. ICD-9 diagnoses, primary-care records, and first-occurrence fields were not used in these finalized OSA definitions. OSA-context models were restricted to participants meeting the corresponding OSA definition; therefore, cases and controls in binary outcome models denote PHQ-2/GAD-2 symptom endpoints rather than OSA status. Three-outcome concordance was calculated only when all three outcome models were estimable; otherwise, concordance was reported for the two estimable MHQ outcomes. Participant-level analysis denominators under all four OSA definitions are summarized in **Supplementary Table 4**, Part A, whereas candidate-level evaluability and directional concordance are summarized in Part B.

### 2.5 Machine-learning candidate selection

To further screen for translational protein candidates from a broad set of proteomic features, we conducted machine-learning-based selection analyses targeting cognitive vulnerability. For this analysis, the cognitive composite was direction-reversed to a cognitive-burden score; participants with burden scores in the highest quartile were defined as cases, while the remaining participants were defined as controls. The merged proteomics data from 16,911 participants were stratified into training (n, 10,146), selection (n, 3,381), and locked-validation (n, 3,384) sets.

We designed 117 prespecified machine-learning workflows, combining 13 feature-selection strategies with 9 classifier types (30–32). Feature-selection methods included strategies based on regularized regression and importance-based tree models. Classifiers included generalized linear models, random forests, radial basis function support vector machines, gradient boosters, glmboost, linear discriminant analysis, naive Bayes, and generalized linear partial least squares regression. Of the 117 prespecified workflows, 103 yielded evaluable performance metrics, whereas 14 failed or were non-evaluable. Thirteen involved xgbTree failures, and one EN_0.8 + svmRadial workflow did not yield evaluable selection-partition probabilities. All 14 remained in the audit denominator. Missing-value handling, standardization, and feature selection were performed using training data only. Formal ranking proceeded by selection AUROC, selection PR-AUC, parsimony, and workflow ID, in that order; locked-validation performance did not influence selection. This workflow was intended to prioritize biologically interpretable protein candidates rather than to develop a clinically deployable proteomic classifier (**Figure 4**; **Supplementary Tables 5B**, **S6**).

**Figure 4:**
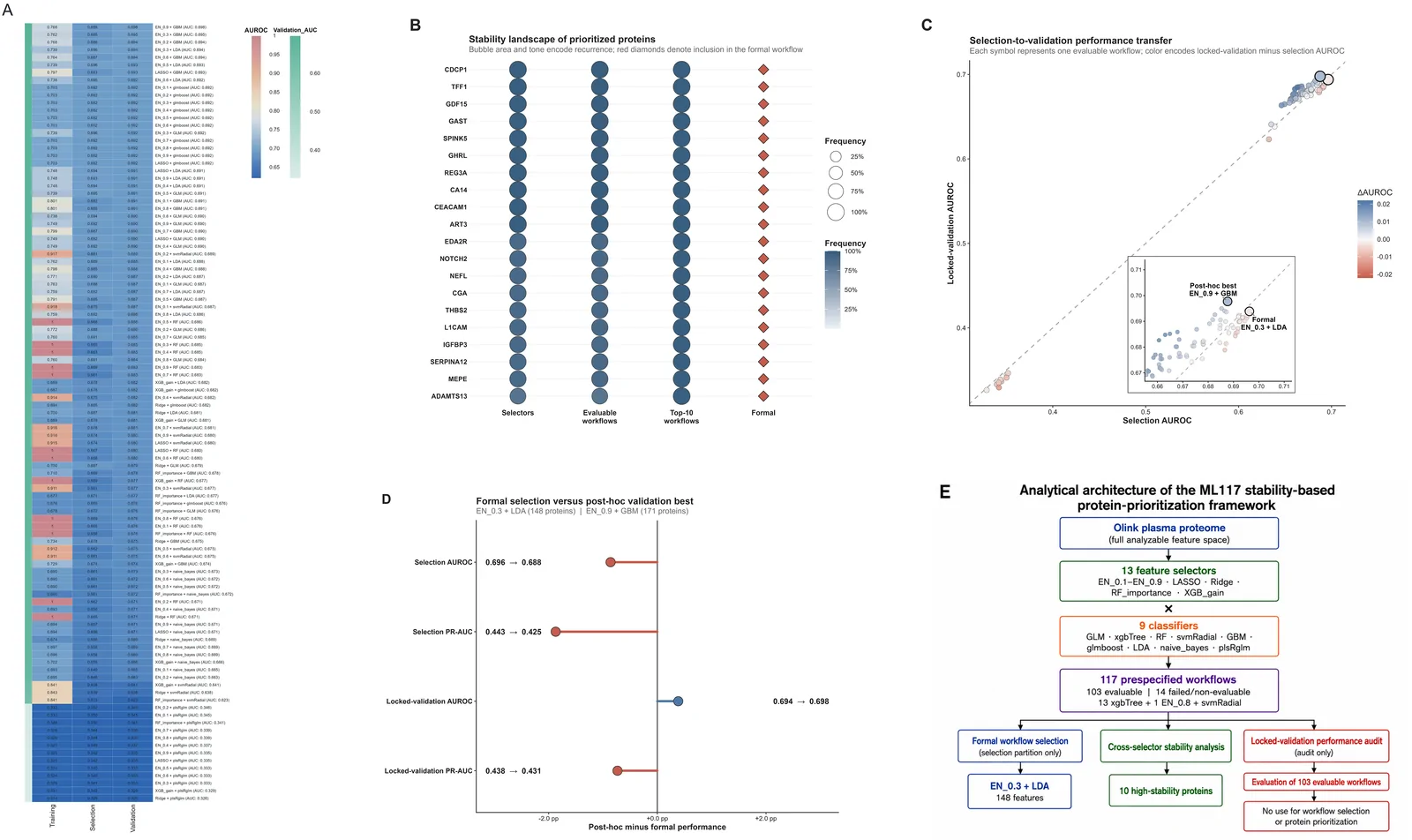
Systematic evaluation of 117 prespecified machine-learning workflows and stability-based proteomic feature prioritization. **(A)** Performance landscape across training, selection, and locked-validation partitions. **(B)** Stability of prioritized protein features across selectors and evaluable workflows; red diamonds indicate inclusion in the formally selected workflow. **(C)** Selection versus locked-validation AUROC, highlighting the formal EN_0.3 + LDA workflow and the *post-hoc* locked-validation-best EN_0.9 + GBM workflow. **(D)** Formal versus *post-hoc* workflows across selection and locked-validation AUROC and PR-AUC. **(E)** Analytical architecture of ML117, including cross-selector stability, formal workflow selection, and locked-validation auditing. AUROC, area under the receiver operating characteristic curve; PR-AUC, area under the precision-recall curve; EN, elastic net; LDA, linear discriminant analysis; GBM, gradient boosting machine.

### 2.6 Depressive-burden risk stratification based on TyG-WHtR

In a separate risk-stratification analysis, we evaluated whether TyG-WHtR could serve as a practical marker for stratifying the burden of depression in OSA. Model development was conducted in the UK Biobank available-source OSA cohort, and clinical evaluation was performed in a cohort of patients with OSA diagnosed by polysomnography (PSG). The primary development cohort used the pre-MHQ available-source OSA definition specified in Section 2.4, based on baseline Data-Field 20002 code 1123 and/or inpatient Data-Field 41270 G47.3 dated by Data-Field 41280 on or before the Data-Field 20400 MHQ completion date. The PHQ-2 outcome was the same MHQ-derived score reconstructed from Data-Fields 20510 and 20514. The primary model retained the full symptom spectrum (PHQ-2 >=4 versus <4; n, 724); comparison of PHQ-2 >=4 with PHQ-2, 0 was retained as an extreme-group sensitivity analysis. The primary clinical-evaluation endpoint was HADS-D >=8, with HADS-D >=11 examined as a sensitivity endpoint; both thresholds were interpreted as symptom-burden categories rather than psychiatric diagnoses. Waist-to-height ratio (WHtR) was calculated as waist circumference (cm)/height (cm), and TyG-WHtR was calculated as TyG × WHtR; equivalently, TyG-WHtR, ln{[fasting triglycerides (mg/dL) × fasting plasma glucose (mg/dL)]/2} × [waist circumference (cm)/height (cm)], consistent with published TyG-WHtR definitions, including prior work on depressive symptoms (52, 65, 66).

Prior to model development, an interpretable logistic regression model was constructed to assess the association with each 1-SD increase in TyG-WHtR, first adjusting for age and sex and then additionally for BMI. Subsequently, restricted cubic spline analysis was used to examine the dose-response relationship between TyG-WHtR and depression burden; results are reported in **Supplementary Table 7**.

Model development began with a core set of metabolic variables, incorporating demographic, physical examination, and surrogate markers of glucose and lipid metabolism and insulin resistance. Subsequently, symptoms, functional, and behavioral variables with cross-setting applicability were gradually added to construct hierarchical regularized logistic regression models (30). Version A added daytime dozing and insomnia to the metabolic core. Version B further included self-rated health, walking pace, and smoking. Version C further incorporated income. Model selection prioritized pooled out-of-fold discrimination, precision-recall performance, and parsimony. Additionally, XGBoost—using the same feature set as Version B—served as the nonlinear comparison model (31). Preprocessing and tuning were conducted within nested resampling folds, and pooled out-of-fold predictions were used to estimate performance and lock the operating threshold, with reporting guided by prediction-model principles (33, 34). Complete results are presented in **Supplementary Table 8**.

The final main model was clinically evaluated outside the UK Biobank in 135 patients with OSA definitively diagnosed via PSG at the Sixth Medical Center of the Chinese PLA General Hospital from September 27, 2025, to April 2, 2026. To support cross-setting evaluation, variables from UK Biobank and single-center clinical data were harmonized. The model, preprocessing steps, and classification threshold locked during development remained unchanged during clinical evaluation. Model performance was evaluated using AUROC, PR-AUC with endpoint prevalence, sensitivity, specificity, positive predictive value, negative predictive value, accuracy, F1 score, and decision-curve analysis. Calibration performance was evaluated using the Brier score, calibration intercept, calibration slope, and calibration plot (33–36). When systematic miscalibration occurred, intercept-only and intercept-plus-slope logistic recalibration were fitted within the same 135-person dataset and treated as apparent *post-hoc* updates, not independent validation (**Figure 5**; **Supplementary Tables 9**, **S11**–**S13**). This recruitment window overlapped with and extended beyond that of the n, 108 deep-phenotyping cohort. Participant-ID cross-checking confirmed that 72 participants (8 women and 64 men) were included in both clinical datasets; therefore, participant-level independence between the two datasets was not assumed, and the n, 135 analysis is described as a single-center clinical evaluation rather than independent external validation.

**Figure 5:**
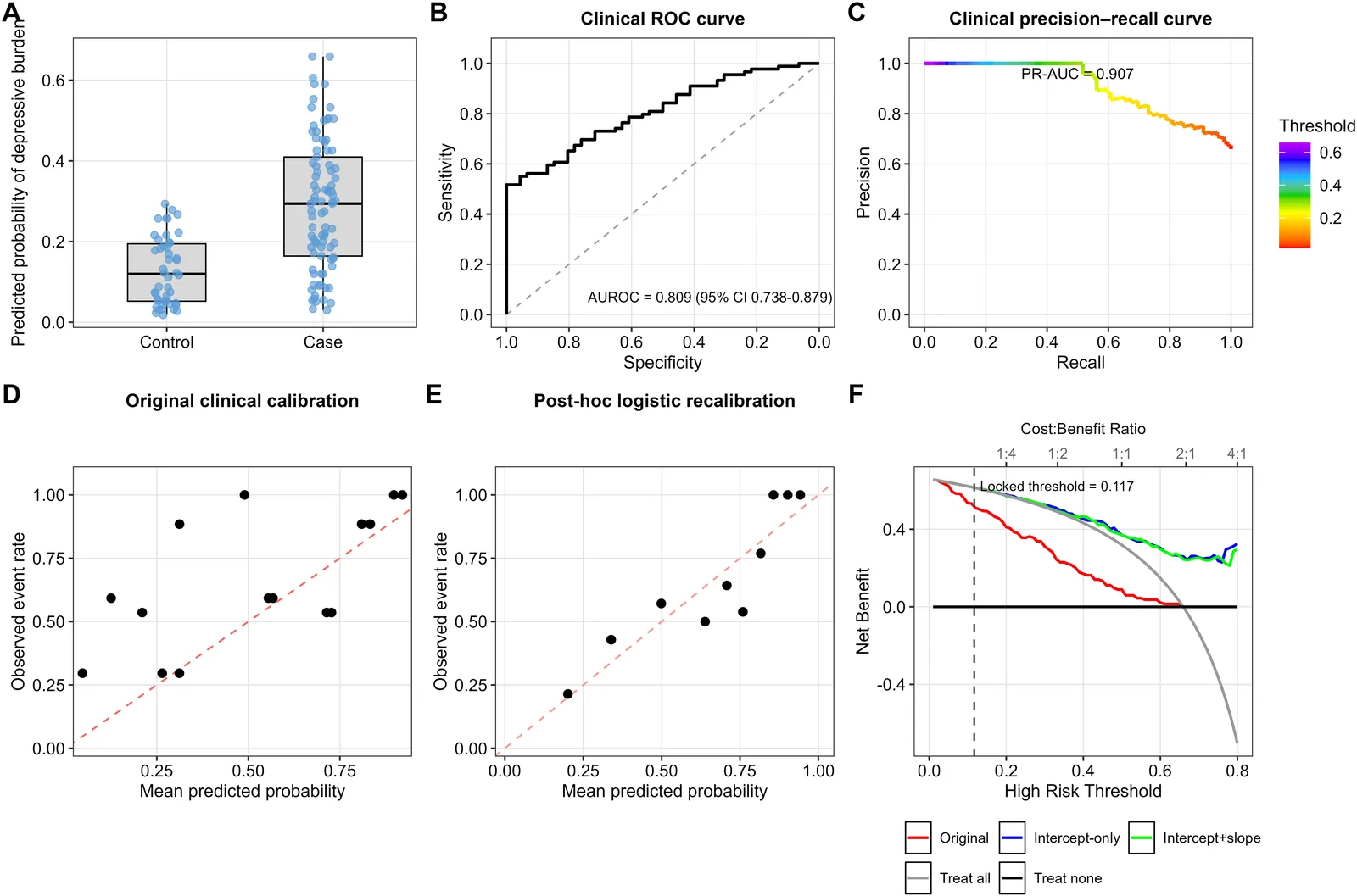
Single-center clinical evaluation of the locked depressive-burden stratification model. **(A)** Distribution of predicted probabilities in clinical controls and cases. **(B)** Clinical ROC curve (AUROC 0.809, 95% CI 0.738-0.879). **(C)** Clinical precision-recall curve (PR-AUC 0.907). **(D)** Original calibration. **(E)** *Post-hoc* logistic recalibration within the same 135-person cohort. **(F)** Decision-curve analysis comparing the locked model, *post-hoc* recalibrated models, and treat-all and treat-none strategies; the vertical line indicates the development-locked threshold of 0.117. The recalibrated curves reflect apparent *post-hoc* analyses and do not constitute independent validation. AUROC, area under the receiver operating characteristic curve; PR-AUC, area under the precision-recall curve; CI, confidence interval.

### 2.7 Human PBMC transcriptomic contextualization

We analyzed the public longitudinal human PBMC RNA-sequencing dataset GSE283703 (BioProject PRJNA1195065), comprising 19 independent participants and 71 PBMC samples. Ten healthy participants contributed 18 control samples, whereas nine participants with OSA contributed 17 untreated-baseline samples, 18 samples after 4 months of CPAP, and 18 samples after 24 months of CPAP. A total of 15,045 genes were retained in the finalized expression analysis. Differential-expression analyses were performed using limma-trend (37). Because repeated samples were available from the same individuals, within-participant correlation was estimated with duplicateCorrelation and incorporated into the linear models; AM/PM sampling time was included as a covariate. Four transcriptome-wide contrasts were prespecified: OSA baseline versus healthy controls, 4-month CPAP versus OSA baseline, 24-month CPAP versus OSA baseline, and 24-month versus 4-month CPAP. Benjamini-Hochberg correction was applied across genes within each contrast, with BH-FDR < 0.05 used to define transcriptome-wide differential expression.

For functional contextualization, Reactome and Gene Ontology Biological Process (GO-BP) analyses were used to summarize pathway-level changes. The predefined 21 circulating-protein candidates were additionally examined at the gene-set level using cameraPR and fgseaMultilevel with multiple-testing correction; candidate-gene effect patterns and longitudinal standardized-expression trajectories were used only as contextual analyses. This PBMC module was prespecified as a human functional-context layer and was not interpreted as independent gene-level validation or direct molecular replication of the circulating proteomic associations (**Figure 6**).

**Figure 6:**
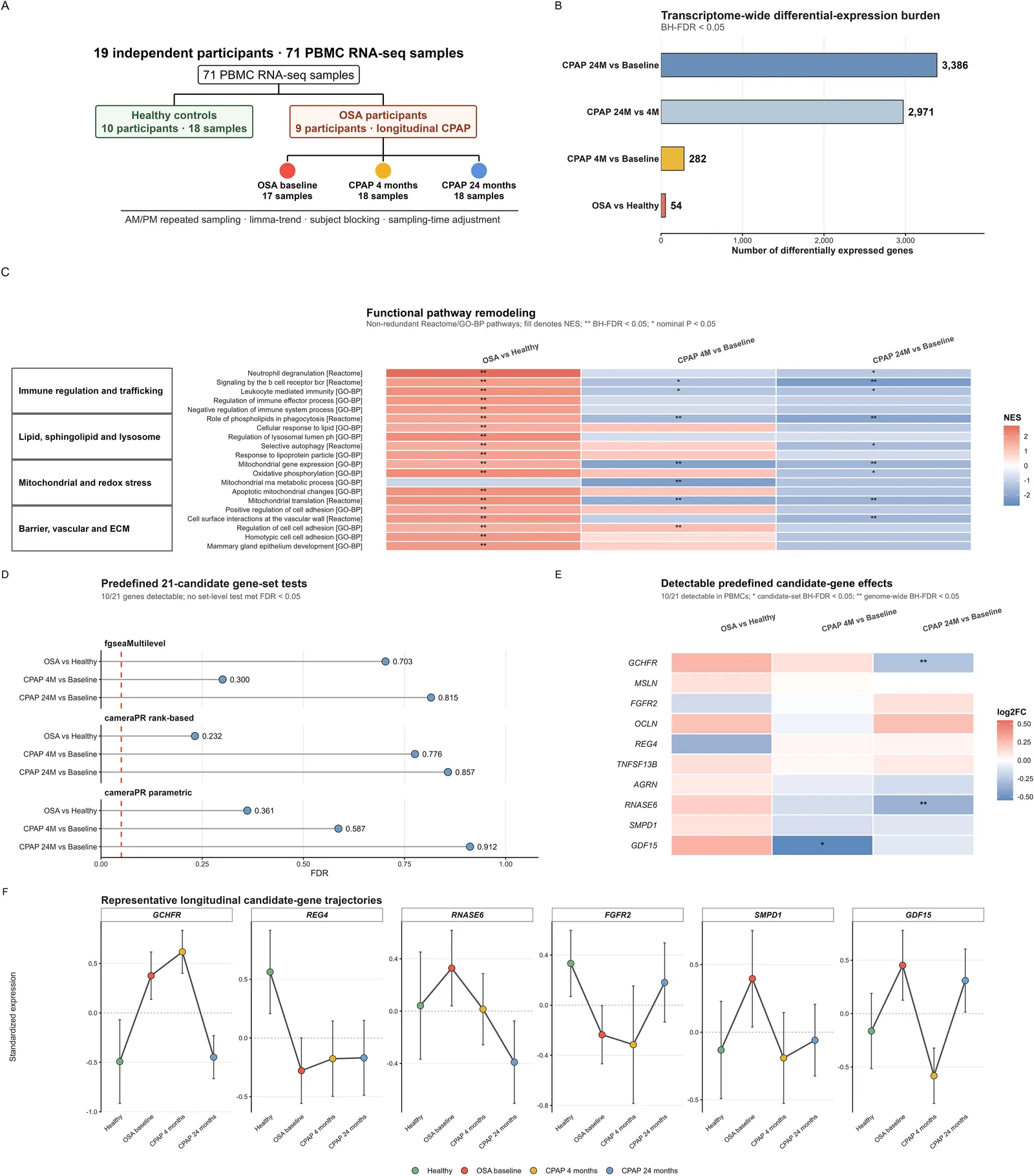
Longitudinal human PBMC transcriptomic contextualization of OSA and CPAP treatment. **(A)** Study design comprising 19 participants and 71 PBMC RNA-sequencing samples across healthy controls, OSA baseline, and 4- and 24-month CPAP follow-up. **(B)** Transcriptome-wide numbers of differentially expressed genes at BH-FDR < 0.05. **(C)** Selected Reactome/GO-BP pathways grouped into immune, lipid/lysosomal, mitochondrial/redox, and vascular/barrier domains. **(D)** Predefined 21-candidate gene-set tests using cameraPR and fgseaMultilevel; no test remained significant after FDR correction. **(E)** Log2 fold-change patterns of the 10 PBMC-detectable candidate genes. **(F)** Longitudinal standardized expression trajectories of representative genes; points show mean ± SEM. OSA, obstructive sleep apnea; CPAP, continuous positive airway pressure; PBMC, peripheral blood mononuclear cell; BH-FDR, Benjamini-Hochberg false discovery rate; GO-BP, Gene Ontology Biological Process; SEM, standard error of the mean.

### 2.8 Statistical analysis

Continuous variables are presented as mean ± standard deviation or median and interquartile range, depending on their distribution; categorical variables are presented as frequency and percentage. Unless otherwise specified, all multivariable models were adjusted for age, sex, and BMI. All statistical tests were two-sided. For multiple comparisons, the Benjamini-Hochberg method was used to control the FDR. For the clinical-evaluation models, both discriminatory power and calibration were evaluated, rather than reporting performance solely from the development phase (35, 36). Analyses used complete cases for the variables required by each prespecified model, with no imputation. No formal *a priori* power calculation was performed; single-center sample sizes reflected all eligible analysis-ready participants within the stated recruitment windows. Model assumptions and influence were examined using heteroscedasticity, specification, Cook-distance, robust-regression, and nonlinear sensitivity analyses where applicable. Since the analyses in this study were conducted within an observational design, conclusions regarding underlying mechanisms were interpreted primarily in terms of biological plausibility, without making causal inferences beyond the available evidence.

## 3 Results

### 3.1 Clinical associations and cross-sectional decomposition in the single-center OSA cohort

The clinical cohort included a total of 108 adult OSA patients; their baseline demographic, sleep, metabolic, and neuropsychological characteristics are shown in **Table 1**. Overall, the cohort was predominantly male, with a mean age of 41 years and a mean AHI of 46 events per hour. Cognitive and emotional burdens were evident at the group level, with a mean MoCA score of 18.9, a mean HADS-A score of 6.39, and a mean HADS-D score of 6.52, as shown in **Table 1**.

**Table 1:** Baseline demographic, polysomnographic, metabolic, and neuropsychological characteristics of the 108-patient single-center deep-phenotyping OSA cohort.

Characteristic	N	Mean ± SD	Median [IQR]
Sex: female	12	12 (11%)	
Sex: male	96	96 (89%)	
Age, years	108	41.31 ± 11.49	39.50 [32.00, 49.00]
BMI, kg/m²	108	28.25 ± 3.76	28.24 [25.39, 30.70]
Apnea-hypopnea index, events/h	108	45.73 ± 27.10	45.40 [21.60, 64.80]
Arousal index, events/h	108	11.32 ± 8.14	10.25 [5.20, 16.22]
TyG index	108	8.93 ± 0.38	9.00 [8.61, 9.07]
MoCA raw score	108	18.85 ± 4.31	18.00 [15.00, 22.00]
Education-corrected MoCA score	108	19.22 ± 4.27	19.00 [15.00, 23.00]
HADS-A score	108	6.39 ± 2.80	6.00 [5.00, 7.00]
HADS-D score	108	6.52 ± 3.07	6.00 [5.00, 8.00]
ESS score	108	7.70 ± 5.88	7.00 [3.00, 10.00]
N3 sleep, %	108	2.49 ± 4.52	0.00 [0.00, 3.50]
Years of education	108	13.40 ± 3.04	14.00 [12.00, 16.00]
HADS-D ≥8, n (%)	108	30 (27.8%)	
HADS-D ≥11, n (%)	108	12 (11.1%)	

Continuous variables are shown as mean ± SD and median [IQR]. MoCA is reported before and after the prespecified education correction. HADS-D thresholds, when available from the analysis-ready dataset, are shown descriptively as symptom-burden categories rather than psychiatric diagnoses. OSA, obstructive sleep apnea; BMI, body mass index; TyG, triglyceride-glucose index; MoCA, Montreal Cognitive Assessment; HADS-A/HADS-D, Hospital Anxiety and Depression Scale anxiety/depression subscales; ESS, Epworth Sleepiness Scale.

Correlation analysis suggested that neuropsychological burden is not a homogeneous construct. Multivariable linear regression further supported this finding. After adjusting for age, sex, and BMI, and additionally for continuous years of education in the MoCA model, both AHI and TyG were significantly and independently associated with cognition. The standardized beta for AHI was -0.540, with a 95% confidence interval of -0.647 to -0.433. The standardized beta for TyG was -0.469, with a 95% confidence interval of -0.609 to -0.328. In comparison, the association between emotional burden and metabolic imbalance was more pronounced. TyG was positively and independently associated with both HADS-A and HADS-D, with standardized betas of 0.550 and 0.568, respectively, whereas AHI and ArI did not demonstrate FDR-significant associations with these two emotional outcomes (**Figure 2A**; **Table 2A**). A 1-SD difference corresponded to approximately 2.3-2.0 MoCA points for AHI-TyG and 1.5-1.7 HADS points for TyG. Sensitivity analyses broadly supported these associations, although the AHI-MoCA spline indicated nonlinearity; the linear beta therefore represents an average association (**Figure 2B**; **Supplementary Table 1**).

**Table 2:** Multivariable associations and exploratory cross-sectional decomposition in the 108-patient deep-phenotyping cohort. Part A. Multivariable associations.

Outcome	Predictor	Standardized beta (95% CI)	P value	BH-FDR	N
Education-corrected MoCA	AHI	-0.540 (-0.647 to -0.433)	<0.001	<0.001	108
Education-corrected MoCA	ArI	-0.087 (-0.211 to 0.037)	0.166	0.213	108
Education-corrected MoCA	TyG	-0.469 (-0.609 to -0.328)	<0.001	<0.001	108
HADS-A	AHI	0.082 (-0.119 to 0.283)	0.419	0.419	108
HADS-A	ArI	0.111 (-0.096 to 0.318)	0.290	0.326	108
HADS-A	TyG	0.550 (0.365 to 0.736)	<0.001	<0.001	108
HADS-D	AHI	0.152 (-0.001 to 0.304)	0.052	0.093	108
HADS-D	ArI	0.117 (-0.039 to 0.274)	0.140	0.210	108
HADS-D	TyG	0.568 (0.372 to 0.764)	<0.001	<0.001	108

AHI, ArI, and TyG were entered simultaneously. All models were adjusted for age, sex, and BMI; the MoCA model additionally accounted for continuous years of education. HC3 heteroscedasticity-consistent standard errors were used. BH-FDR was calculated across the nine primary predictor-outcome tests.

Exploratory cross-sectional decomposition revealed that the ordering from AHI to TyG to education-corrected MoCA was primarily driven by the direct statistical component, with no significant indirect component. In contrast, the ordering from ArI to TyG to HADS-A was dominated by an indirect statistical component, while the direct component was not significant. The ordering from ArI to TyG to HADS-D likewise retained a significant indirect component and a nonsignificant direct component; reverse-order indirect components were not significant (**Figure 2C**; **Table 2B**; **Supplementary Table 1**). Competing-exposure adjustment did not alter the reported indirect-component significance pattern; the corresponding exposure-outcome and exposure-TyG regressions are reported in **Supplementary Table 1B**, Part A. In sensitivity decompositions expressed in outcome-score units per 1-SD higher exposure, the AHI -> TyG -> education-corrected MoCA indirect statistical component remained nonsignificant after additional ArI adjustment (0.241, 95% bootstrap CI -0.090 to 0.656; P, 0.154), whereas the direct statistical component remained significant (-2.307, 95% bootstrap CI -2.751 to -1.849; P < 0.001). After additional AHI adjustment, the indirect statistical components remained significant for ArI -> TyG -> HADS-A (0.626, 95% bootstrap CI 0.305 to 1.031; P < 0.001) and ArI -> TyG -> HADS-D (0.708, 95% bootstrap CI 0.356 to 1.152; P < 0.001) (**Supplementary Table 1B**, Part B). These results are consistent with partly different association patterns but do not establish temporal mediation or distinct causal pathways; TyG is better viewed as a parallel cognitive correlate.

**Table 2:** Part B. Exploratory cross-sectional association decomposition.

Prespecified ordering	Component	Estimate (95% bootstrap CI)	Bootstrap P	N
AHI → TyG → education-corrected MoCA	Indirect statistical component	0.056 (-0.024 to 0.159)	0.148	108
AHI → TyG → education-corrected MoCA	Direct statistical component	-0.540 (-0.640 to -0.433)	<0.001	108
AHI → TyG → education-corrected MoCA	Total association	-0.483 (-0.619 to -0.334)	<0.001	108
ArI → TyG → HADS-A	Indirect statistical component	0.219 (0.106 to 0.372)	<0.001	108
ArI → TyG → HADS-A	Direct statistical component	0.111 (-0.088 to 0.292)	0.306	108
ArI → TyG → HADS-A	Total association	0.330 (0.123 to 0.541)	0.004	108
ArI → TyG → HADS-D	Indirect statistical component	0.226 (0.116 to 0.376)	<0.001	108
ArI → TyG → HADS-D	Direct statistical component	0.117 (-0.045 to 0.272)	0.147	108
ArI → TyG → HADS-D	Total association	0.344 (0.167 to 0.542)	<0.001	108

All variables were measured within the same cross-sectional clinical assessment. The indirect/direct decomposition used 5,000 nonparametric bootstrap samples and is interpreted only as an associational decomposition. No temporal sequence or causal mediation is inferred. Reverse-order analyses are reported in **Supplementary Table 1**.

### 3.2 General-population cross-outcome proteomic associations

In the UK Biobank Olink proteomics subsets, the source sample size was 16,814 for the cognitive composite endpoint COG_z, 16,454 for the PHQ-2 depression endpoint, and 16,428 for the GAD-2 anxiety endpoint. Among 2,923 QC-analyzable proteins, 2,922 were estimable for each outcome. Protein-specific sample sizes varied, with median n, 14,412, 13,630, and 13,612, respectively. After full-proteome FDR correction, 886 proteins were significantly associated with cognition, 152 with depression, and 45 with anxiety. A total of 21 proteins overlapped across all three endpoints (**Figure 3A**; **Supplementary Table 2**).

All 21 proteins showed the adverse-direction pattern, but effects were modest: cognition beta -0.105 to -0.016, PHQ-2 odds ratio 1.131-1.254, and GAD-2 odds ratio 1.118-1.173 per 1-SD NPX (**Figure 3C**; **Supplementary Table 2**). In the strict-adjustment common complete-case sample (n, 5,296; complete data for the three outcomes, CRP, and SII), PHQ-2 and GAD-2 were moderately correlated (Pearson r, 0.573), whereas cognition showed weak correlations with PHQ-2 and GAD-2 (r, -0.038 and -0.065, respectively). Correlation-preserving permutation and missingness-matched random-set analyses yielded empirical P, 0.00020 and P, 0.00010, respectively (**Figure 3B**; **Supplementary Table 3**). After strict mutual-outcome and CRP/SII adjustment, 2, 2, and 0 proteins remained FDR-significant for cognition, PHQ-2, and GAD-2, with none significant across two or more outcomes (**Supplementary Table 3**). In the ever-recorded available-source OSA subset, 8/21 proteins were concordant for all three outcomes and 18/21 for at least two; in the pre-MHQ available-source OSA subset, 14/21 were concordant for PHQ-2 and GAD-2, while cognition was not estimable. No individual OSA-context association survived FDR correction (**Supplementary Table 4**); however, the retained directional concordance provides preliminary evidence of consistency within these smaller OSA subsets. Because the OSA-context analyses were based on substantially smaller subsets than the general-population analyses, limited statistical precision may have reduced power to detect modest effects after multiple-testing correction. These findings therefore support further validation in larger, deeply phenotyped OSA proteomic cohorts rather than a definitive negative replication.

### 3.3 Systematic machine-learning protein-feature prioritization

To identify more actionable candidate proteins from a broad set of proteomic features, we conducted machine-learning-based selection analyses targeting cognitive vulnerability. A total of 117 predefined workflows were tested, of which 103 yielded evaluable performance metrics and 14 failed or were non-evaluable. The formally selected EN_0.3 + LDA workflow retained 148 features and achieved AUROC values of 0.696 and 0.694 on the selection and locked-validation sets, respectively, with locked-validation PR-AUC 0.438 and Brier score 0.172 (**Figure 4**; **Supplementary Tables 5B**, **S6**). Based on this framework, the candidate proteins were interpreted as exploratory priorities rather than as a clinical classifier or validation of the 21-protein set.

### 3.4 TyG-WHtR and depressive-symptom burden risk stratification

In the separate risk-stratification analysis, we evaluated whether TyG-WHtR could serve as a candidate marker for stratifying the burden of depression in OSA. In the UK Biobank full-spectrum available-source OSA cohort, the age- and sex-adjusted odds ratio per 1-SD higher TyG-WHtR was 1.779 (95% CI 1.332-2.376; n, 620), but this association attenuated to 1.370 after BMI adjustment (95% CI 0.810-2.319; P, 0.241; n, 619; **Table 3 Part A**). Restricted cubic spline analysis showed no strong nonlinear component. These results do not support an association independent of overall adiposity (**Supplementary Table 7**).

**Table 3:** Development, clinical evaluation, and calibration of the depressive-symptom burden risk-stratification model. Part A. TyG-WHtR association in available-source OSA.

Label strategy	Adjustment	N (cases/controls)	OR per 1-SD TyG-WHtR (95% CI)	P value
Full spectrum	Age + sex	620 (48/572)	1.779 (1.332 to 2.376)	<0.001
Full spectrum	Age + sex + BMI	619 (48/571)	1.370 (0.810 to 2.319)	0.241
Strict sensitivity	Age + sex	404 (48/356)	2.161 (1.564 to 2.986)	<0.001
Strict sensitivity	Age + sex + BMI	403 (48/355)	1.479 (0.810 to 2.700)	0.203

Effects are reported per 1-SD higher TyG-WHtR. Full-spectrum analyses retain intermediate PHQ-2 symptom levels; the strict extreme-group analysis is a sensitivity analysis.

Within the hierarchical modeling framework, Version B was selected as the primary model because it achieved the optimal balance between discriminatory power, precision-recall performance, and model parsimony. In the full-spectrum cohort of 724 participants (63 cases; prevalence 8.7%), the pooled nested out-of-fold AUROC was 0.726, the PR-AUC was 0.236, and the Brier score was 0.075. At the locked threshold of 0.117, sensitivity was 0.587 and specificity was 0.809 (**Table 3B**; **Supplementary Table 8**).

**Table 3:** Part B. Primary full-spectrum UKB model: nested out-of-fold performance.

Item	Value	95% bootstrap CI
N	724	
Cases	63	
Event prevalence	8.7%	
Nested OOF AUROC	0.726	0.645 to 0.801
Nested OOF PR-AUC	0.236	0.158 to 0.338
Brier score	0.075	0.061 to 0.090
Calibration intercept	-0.505	-1.461 to 0.491
Calibration slope	0.769	0.378 to 1.242
Locked OOF threshold	0.117	
Sensitivity	0.587	0.463 to 0.709
Specificity	0.809	0.778 to 0.837
False-positive rate	0.191	0.163 to 0.222

The primary model is the locked Version B elastic-net model in the pre-MHQ available-source OSA cohort. Preprocessing and tuning were estimated within resampling folds. The operating threshold was locked from pooled nested out-of-fold predictions.

### 3.5 Clinical evaluation and recalibration

The Version B model developed using UK Biobank data was evaluated in 135 patients with OSA definitively diagnosed by PSG from the Sixth Medical Center of the Chinese PLA General Hospital (89 HADS-D >=8 cases; prevalence 0.659). The clinical-evaluation AUROC was 0.809 (95% CI 0.738-0.879), and the PR-AUC was 0.907. At the unchanged cutoff of 0.117, sensitivity was 0.843, specificity was 0.500, and the false-positive rate 0.500 (**Figures 5A-C**; **Table 3C**; **Supplementary Table 9**). The PR-AUC should be interpreted relative to the high endpoint prevalence; these data support ranking, not independent validation.

**Table 3:** Part C. Single-center clinical evaluation and apparent *post-hoc* recalibration.

Model/update	N (cases/controls)	AUROC (95% CI)	PR-AUC	Brier	Calibration intercept	Calibration slope	Sensitivity	Specificity	False-positive rate
Original locked model	135 (89/46)	0.809 (0.738 to 0.879)	0.907	0.356	2.556	1.181	0.843	0.500	0.500
*Post-hoc* intercept-only update	135 (89/46)	0.809 (0.738 to 0.879)	0.907	0.173	-0.100	1.181	1.000	0.000	1.000
*Post-hoc* intercept+slope update	135 (89/46)	0.809 (0.738 to 0.879)	0.907	0.174	0.000	1.000	1.000	0.022	0.978

The clinical evaluation used 135 single-center OSA participants and a HADS-D-based symptom-burden endpoint. *Post-hoc* recalibration is an apparent local update and is not independent validation. Discrimination should be interpreted separately from absolute-risk calibration.

On the other hand, the model performed poorly in calibration within the clinical-evaluation cohort (Brier score 0.356; calibration intercept 2.556; slope 1.181; **Figure 5D**; **Table 3C**). Following same-cohort *post-hoc* recalibration, the consistency between predicted probabilities and actual risks improved, while the AUROC and PR-AUC remained unchanged; these estimates were not independently validated (**Figures 5E, F**; **Supplementary Table 12**). Decision-curve analysis showed no clear net-benefit advantage at the locked threshold. Overall, the model retained encouraging cross-setting risk-ranking performance, whereas absolute probability estimates were less stable and may require local recalibration before prospective clinical use.

### 3.6 Longitudinal human PBMC transcriptomic contextualization

Among 15,045 genes, the four comparisons yielded 54, 282, 3,386, and 2,971 differentially expressed genes, respectively (BH-FDR < 0.05; **Figures 6A-C**). Pathway-level analyses highlighted immune, lipid/lysosomal, mitochondrial/redox, and vascular/barrier processes across OSA and CPAP-related states, providing human functional context for systemic inflammatory, metabolic, redox, and vascular responses in OSA (**Figures 6A-C**). The predefined 21-gene analysis did not remain FDR-significant and was therefore treated as contextual rather than direct molecular replication (**Figures 6D-F**).

## 4 Discussion

This study reveals the multidimensional heterogeneity of neuropsychological vulnerability in OSA and establishes a complementary framework with clearly differentiated levels of evidence across clinical phenotyping, circulating proteomics, functional transcriptomics, and risk stratification. At the clinical level, cognitive and affective burdens did not vary uniformly with the severity of respiratory events: cognition was associated with both respiratory and metabolic burden, whereas affective burden showed a stronger association with metabolic dysregulation. Further cross-sectional decomposition demonstrated significant indirect statistical components for both the ArI→TyG→HADS-A and ArI→TyG→HADS-D orderings, which remained robust after adjustment for AHI as a competing exposure. These findings suggest that sleep-fragmentation-related metabolic dysregulation may represent an important clinical context for understanding affective vulnerability in OSA, although the cross-sectional nature of the data precludes inference of temporal mediation or causal pathways. Consistent with this clinical heterogeneity, full-proteome analysis in the general population identified 21 cross-outcome proteins with non-random adverse-direction concordance, supporting a distributed immunometabolic, redox, and vascular biological background rather than convergence on a single OSA-specific molecular axis. Building on these complementary evidence layers, we further applied prespecified machine-learning workflows for protein-feature prioritization and evaluated the potential of TyG-WHtR and routinely available clinical variables for depressive-burden risk stratification. Clinical evaluation indicated that cross-setting risk ranking was relatively stable, whereas absolute probability estimates remained substantially calibration-dependent.

The OSA clinical cohort from the Sixth Medical Center of the Chinese PLA General Hospital provides direct support for the aforementioned differentiated clinical association pattern rather than for a causal mechanism. After multivariable adjustment, both AHI and TyG were independently associated with cognitive performance, whereas affective burden was more strongly associated with TyG. The cross-sectional decomposition further showed significant indirect statistical components for the ArI -> TyG -> HADS-A and ArI -> TyG -> HADS-D orderings, whereas the AHI -> TyG -> MoCA indirect component was not significant; reverse-order and competing-exposure analyses were used to probe the stability of these patterns. Because all variables were measured cross-sectionally and the estimates depended on the imposed ordering, these findings cannot establish temporal mediation. Our results suggest that the different pathophysiological components of OSA are not uniformly associated with neuropsychological outcomes but show associations of varying magnitudes across cognitive and affective dimensions, with sleep-fragmentation-related metabolic imbalance potentially being particularly relevant to affective burden, although a distinct causal pathway cannot be inferred. From a biological perspective, this pattern is consistent with respiratory burden, sleep fragmentation, and metabolic disturbances—factors that represent biologically independent components of OSA-related stress, rather than merely the severity of respiratory obstruction alone. Clinically, this supports a multidimensional phenotypic analysis, in which AHI is supplemented with information from metabolic and sleep fragmentation measures when assessing neuropsychological vulnerability in patients with OSA—particularly in identifying those whose neuropsychological vulnerability cannot be adequately reflected by respiratory severity alone (38–43).

The proteomic-level findings provide broader general-population systems-biology context for the aforementioned clinical heterogeneity. We analyzed the full 2,923-protein panel under a uniform proteome-wide FDR framework rather than restricting the analysis to a candidate subset. A total of 21 proteins were associated with cognition, depression, and anxiety simultaneously, and their adverse-direction concordance exceeded post-selection null expectations. These overlapping proteins showed clear directional consistency, but the individual effect sizes were modest. PHQ-2 and GAD-2 were moderately correlated, whereas cognition was only weakly correlated with either affective outcome; therefore, the 21-protein overlap cannot be explained simply by strong correlation among all three neuropsychological phenotypes. The combination of small individual effects and non-random collective concordance is more compatible with a distributed, multi-protein systemic susceptibility background than with a single high-effect circulating biomarker. Attenuation after mutual-outcome and CRP/SII adjustment further indicates that part of the overlap is shared with common symptom and inflammatory burden. In OSA-context analyses, directional consistency remained common but no individual association survived FDR correction, which is compatible with limited precision in the substantially smaller OSA subsets and supports further evaluation in larger PSG-phenotyped cohorts. Consequently, OSA-related neuropsychological vulnerability may reflect a broader systemic susceptibility context rather than a clinical phenotype defined solely by the burden of respiratory events. Together with prior evidence implicating endothelial, inflammatory, hypoxia-related, and complement-related processes (38–45), these findings remain biologically plausible while stopping short of an OSA-specific molecular mechanism.

The value of machine learning-based screening in this study lies not in building directly applicable proteomic prediction tools, but in identifying a smaller, more testable set of priority molecules from a high-dimensional proteomic search space. The formal workflow showed similar selection and locked-validation performance, and proteins repeatedly prioritized across different feature-selection strategies and evaluable workflows may represent signals with some robustness to the specific algorithmic choice. They are therefore better viewed as candidates for downstream experimental validation than as established mechanistic proteins. The longitudinal human PBMC analysis adds a complementary functional layer: immune, lipid/lysosomal, mitochondrial/redox, and vascular/barrier processes were repeatedly represented across OSA and CPAP-related states. At the functional level, these domains show contextual convergence with the clinical metabolic associations and with the inflammatory, metabolic, vascular, and complement-related background suggested by the circulating proteomic findings. This functional-level biological consistency is informative.

Our translational analysis further enhances the clinical interpretability of the above results. TyG-WHtR was associated with depressive-symptom burden after adjustment for age and sex, but the association attenuated after additional BMI adjustment. This attenuation suggests that the signal captured by TyG-WHtR overlaps, at least in part, with broader adiposity-related metabolic burden rather than representing a biological axis independent of overall adiposity. The dose-response analysis did not identify a strong nonlinear component. Prior studies have linked TyG or related indices with depressive symptoms, OSA, and sleep-disorder phenotypes (46–59), while associations with cerebrovascular and coronary disease illustrate the broader, nonspecific cardiometabolic context of TyG (60, 61). Furthermore, the main model developed using UK Biobank data demonstrated encouraging discrimination in the single-center PSG cohort, supporting cross-setting risk ranking without establishing stable transportability of absolute probabilities. Within this clinical-evaluation dataset, the model’s absolute risk estimates showed substantial calibration deviation; after same-cohort *post-hoc* recalibration, the consistency between predicted probabilities and actual risks improved, while discriminatory performance remained largely stable. These findings suggest that the model may be more useful for risk enrichment to prioritize further neuropsychological assessment than as a stand-alone probability-based decision tool. Local probability adjustment appears feasible, but its value must be confirmed prospectively in a genuinely new cohort. Our findings also suggest an interesting pattern: neuropsychological heterogeneity in OSA is closely associated with a broader systemic inflammatory and metabolic context, consistent with previous evidence that routinely available biomarkers such as CRP, SII, and fibrinogen may provide complementary and low-cost inflammatory phenotyping (62). Future studies should further integrate conventional polysomnography (PSG)-defined disease severity with metabolic and inflammatory biomarkers and upper-airway anatomical phenotyping; previous studies in surgical OSA cohorts have also demonstrated the clinical relevance of anatomy-based patient selection (63). In addition, physiological traits and longitudinal treatment responses should be prospectively evaluated as complementary dimensions of multidimensional patient phenotyping.

This study has certain limitations. Both the single-center clinical cohort and the UK Biobank analyses are observational; therefore, we cannot infer a causal relationship between OSA-related metrics, such as AHI, ArI, and TyG, and patients’ neuropsychological burden. The primary role of the machine-learning workflow is exploratory proteomic feature prioritization and improved interpretability, rather than the development of a definitive clinical proteomic prediction tool. The proteomic analysis used general-population outcomes, the null analyses were performed after selection of the 21-protein set, and the PBMC analysis provided functional context rather than molecular replication. The translational model retained the full PHQ-2 symptom spectrum in development but used HADS-D in clinical evaluation. In addition, participant-ID cross-checking identified 72 shared participants (8 women and 64 men) between the n, 108 deep-phenotyping cohort and the n, 135 clinical-evaluation dataset; accordingly, the latter should not be interpreted as participant-independent external validation. Both single-center clinical datasets were predominantly male, limiting female-specific inference. Male-restricted, sex-interaction, and sex-stratified clinical-evaluation analyses are therefore presented as sensitivity analyses in **Supplementary Tables 1**, **S7**, and **S10**, and sex-specific findings should be interpreted cautiously given the limited number of women. Overall, these factors primarily define the scope of the current conclusions rather than undermining the key biological directions revealed by this study.

Nevertheless, this study offers distinct advantages. We examined clinically observed neuropsychological heterogeneity alongside general-population proteomic context and, through systematic candidate prioritization and longitudinal human PBMC analysis, retained biological interpretation without treating these layers as direct validation. Compared with studies confined to proteomic screening without clinical extension, or those emphasizing model performance without biological context, this study integrates complementary evidence layers spanning clinical phenotypes, molecular characteristics, and translational applications. Its significance lies not in proposing another set of OSA biomarkers, but in supporting a multidimensional theoretical hypothesis in which respiratory severity, metabolic status, and systemic inflammatory/vascular context may be jointly associated with neuropsychological vulnerability, while clinically accessible metabolic information may assist risk enrichment without being treated as a stand-alone diagnostic signal. Taken together, cross-layer correspondence was observed primarily at the level of biological function rather than individual molecules, linking differentiated clinical phenotypes to a distributed systemic susceptibility context involving metabolic, inflammatory, redox, and vascular processes.

## 5 Conclusions

This study demonstrates that neuropsychological vulnerability in OSA is multidimensional and cannot be explained solely by the severity of respiratory events reflected by the AHI. Cognitive performance was associated with both respiratory and metabolic burden, whereas sleep-fragmentation-related metabolic dysregulation may be more closely related to affective symptom burden, suggesting that respiratory burden, sleep fragmentation, and metabolic dysregulation represent interconnected but distinct clinical dimensions. The research results of circulating proteomics and human peripheral blood mononuclear cell transcriptomics further support that the neuropsychological heterogeneity in patients with OSA might be due to a broader systemic background involving immune metabolism, redox processes, and vascular biological processes. The depressive-burden model also demonstrated the potential of integrating clinically accessible metabolic and phenotypic information for cross-setting risk stratification. Overall, our findings support expanding OSA neuropsychological risk assessment beyond an AHI-centered, unidimensional severity framework toward multidimensional phenotyping that incorporates respiratory burden, sleep fragmentation, metabolic status, and the broader systemic biological context.

## Data Availability

The original contributions presented in the study are included in the article/**Supplementary Material**. Further inquiries can be directed to the corresponding authors.
