# Supplementary material for: Multidimensional neuropsychological heterogeneity in obstructive sleep apnea: deep clinical phenotyping in a single-center cohort, population proteomic context, and depressive-burden risk stratification
Source: Front Immunol. 2026 Sep 15;17:1874192. doi: 10.3389/fimmu.2026.1874192 (PMC13619841; doi:10.3389/fimmu.2026.1874192)
Supplement: Supplementary file 1 [file Supplementaryfile1.docx]

**Supplementary Table S1. Sensitivity and robustness analyses for the 108-patient deep-phenotyping cohort**

**Part A. Education adjustment and MoCA sensitivity**

| **Model** | **Predictor** | **Beta (95% CI)** | **P** | **n** |
| --- | --- | --- | --- | --- |
| Raw MoCA, no education covariate | AHI | -0.534 (-0.641 to -0.428) | <0.001 | 108 |
| Raw MoCA, no education covariate | ArI | -0.079 (-0.200 to 0.041) | 0.193 | 108 |
| Raw MoCA, no education covariate | TyG | -0.458 (-0.597 to -0.319) | <0.001 | 108 |
| Education-corrected MoCA, no continuous education covariate | AHI | -0.532 (-0.642 to -0.422) | <0.001 | 108 |
| Education-corrected MoCA, no continuous education covariate | ArI | -0.085 (-0.210 to 0.041) | 0.184 | 108 |
| Education-corrected MoCA, no continuous education covariate | TyG | -0.439 (-0.585 to -0.292) | <0.001 | 108 |
| Education-corrected MoCA + continuous education covariate | AHI | -0.540 (-0.647 to -0.433) | <0.001 | 108 |
| Education-corrected MoCA + continuous education covariate | ArI | -0.087 (-0.211 to 0.037) | 0.166 | 108 |
| Education-corrected MoCA + continuous education covariate | TyG | -0.469 (-0.609 to -0.328) | <0.001 | 108 |

Note: Comparison of raw versus education-corrected MoCA specifications.

**Part B. Model diagnostics**

| **Outcome** | **n** | **Maximum VIF** | **BP P** | **RESET P** | **Cook flagged, n** | **Maximum Cook distance** |
| --- | --- | --- | --- | --- | --- | --- |
| Education-corrected MoCA | 108 | 1.580 | 0.406 | 0.003 | 10 | 0.064 |
| HADS-A | 108 | 1.580 | 0.078 | 0.556 | 6 | 0.204 |
| HADS-D | 108 | 1.580 | 0.024 | 0.008 | 4 | 0.105 |

Note: BP = Breusch-Pagan test; RESET = regression specification error test.

**Part C. Huber and Cook-distance sensitivity analyses**

| **Outcome** | **Analysis** | **Predictor** | **n** | **Beta (95% CI)** | **P** |
| --- | --- | --- | --- | --- | --- |
| Education-corrected MoCA | Cook-distance-deleted | AHI | 98 | -0.572 (-0.668 to -0.477) | <0.001 |
| Education-corrected MoCA | Cook-distance-deleted | ArI | 98 | -0.078 (-0.192 to 0.036) | 0.179 |
| Education-corrected MoCA | Cook-distance-deleted | TyG | 98 | -0.432 (-0.538 to -0.326) | <0.001 |
| Education-corrected MoCA | Huber robust regression | AHI | 108 | -0.550 (-0.671 to -0.429) | <0.001 |
| Education-corrected MoCA | Huber robust regression | ArI | 108 | -0.071 (-0.209 to 0.066) | 0.308 |
| Education-corrected MoCA | Huber robust regression | TyG | 108 | -0.468 (-0.592 to -0.344) | <0.001 |
| HADS-A | Cook-distance-deleted | AHI | 102 | 0.084 (-0.074 to 0.242) | 0.293 |
| HADS-A | Cook-distance-deleted | ArI | 102 | -0.044 (-0.204 to 0.117) | 0.589 |
| HADS-A | Cook-distance-deleted | TyG | 102 | 0.527 (0.346 to 0.707) | <0.001 |
| HADS-A | Huber robust regression | AHI | 108 | 0.018 (-0.143 to 0.179) | 0.824 |
| HADS-A | Huber robust regression | ArI | 108 | 0.056 (-0.126 to 0.239) | 0.545 |
| HADS-A | Huber robust regression | TyG | 108 | 0.548 (0.383 to 0.713) | <0.001 |
| HADS-D | Cook-distance-deleted | AHI | 104 | 0.169 (0.031 to 0.306) | 0.017 |
| HADS-D | Cook-distance-deleted | ArI | 104 | 0.157 (0.024 to 0.290) | 0.021 |
| HADS-D | Cook-distance-deleted | TyG | 104 | 0.617 (0.468 to 0.765) | <0.001 |
| HADS-D | Huber robust regression | AHI | 108 | 0.143 (-0.002 to 0.289) | 0.054 |
| HADS-D | Huber robust regression | ArI | 108 | 0.145 (-0.020 to 0.311) | 0.085 |
| HADS-D | Huber robust regression | TyG | 108 | 0.582 (0.433 to 0.732) | <0.001 |

Note: Robustness analyses assessed heteroscedasticity and influential observations.

**Part D. Nonlinearity and support/influence audit**

| **Outcome** | **Exposure** | **n** | **Spline BH-FDR** | **Central-support BH-FDR** | **Cook-deleted BH-FDR** | **Interpretation** |
| --- | --- | --- | --- | --- | --- | --- |
| Education-corrected MoCA | AHI | 108 | 0.020 | <0.001 | 0.010 | Consistent evidence of nonlinearity |
| Education-corrected MoCA | ArI | 108 | 0.051 | 0.110 | 0.010 | Sensitivity-only nonlinearity; do not override primary linear model |
| Education-corrected MoCA | TyG | 108 | 0.715 | 0.108 | 0.053 | No BH-supported nonlinearity |
| HADS-A | AHI | 108 | 0.143 | 0.582 | 0.588 | No BH-supported nonlinearity |
| HADS-A | ArI | 108 | 0.088 | 0.595 | <0.001 | Sensitivity-only nonlinearity; do not override primary linear model |
| HADS-A | TyG | 108 | 0.143 | 0.110 | 0.026 | Sensitivity-only nonlinearity; do not override primary linear model |
| HADS-D | AHI | 108 | 0.313 | 0.582 | 0.096 | No BH-supported nonlinearity |
| HADS-D | ArI | 108 | 0.024 | 0.110 | 0.010 | Full-sample signal not robust to support/influence audit |
| HADS-D | TyG | 108 | 0.125 | 0.582 | 0.255 | No BH-supported nonlinearity |

Note: Nonlinear findings were interpreted only after multiplicity correction and support/influence sensitivity analyses.

**Part E. Original and reverse-order cross-sectional association decompositions**

| **Ordering** | **Component** | **n** | **Estimate (95% bootstrap CI)** | **P** |
| --- | --- | --- | --- | --- |
| AHI_to_TyG_to_MoCA | Indirect | 108 | 0.056 (-0.024 to 0.159) | 0.148 |
| AHI_to_TyG_to_MoCA | Direct | 108 | -0.540 (-0.640 to -0.433) | <0.001 |
| AHI_to_TyG_to_MoCA | Total | 108 | -0.483 (-0.619 to -0.334) | <0.001 |
| AHI_to_TyG_to_MoCA | Proportion | 108 | -0.117 (-0.433 to 0.042) | 0.148 |
| AHI_to_TyG_to_MoCA | a path | 108 | -0.120 (-0.322 to 0.050) | 0.148 |
| AHI_to_TyG_to_MoCA | b path | 108 | -0.469 (-0.617 to -0.332) | <0.001 |
| TyG_to_AHI_to_MoCA_reverse | Indirect | 108 | 0.068 (-0.030 to 0.165) | 0.171 |
| TyG_to_AHI_to_MoCA_reverse | Direct | 108 | -0.469 (-0.623 to -0.337) | <0.001 |
| TyG_to_AHI_to_MoCA_reverse | Total | 108 | -0.400 (-0.580 to -0.239) | <0.001 |
| TyG_to_AHI_to_MoCA_reverse | Proportion | 108 | -0.171 (-0.600 to 0.059) | 0.171 |
| TyG_to_AHI_to_MoCA_reverse | a path | 108 | -0.127 (-0.301 to 0.055) | 0.171 |
| TyG_to_AHI_to_MoCA_reverse | b path | 108 | -0.540 (-0.641 to -0.438) | <0.001 |
| ArI_to_TyG_to_HADS_A | Indirect | 108 | 0.219 (0.106 to 0.372) | <0.001 |
| ArI_to_TyG_to_HADS_A | Direct | 108 | 0.111 (-0.088 to 0.292) | 0.306 |
| ArI_to_TyG_to_HADS_A | Total | 108 | 0.330 (0.123 to 0.541) | 0.004 |
| ArI_to_TyG_to_HADS_A | Proportion | 108 | 0.663 (0.345 to 1.524) | 0.004 |
| ArI_to_TyG_to_HADS_A | a path | 108 | 0.398 (0.234 to 0.596) | <0.001 |
| ArI_to_TyG_to_HADS_A | b path | 108 | 0.550 (0.342 to 0.722) | <0.001 |
| TyG_to_ArI_to_HADS_A_reverse | Indirect | 108 | 0.036 (-0.030 to 0.114) | 0.306 |
| TyG_to_ArI_to_HADS_A_reverse | Direct | 108 | 0.550 (0.342 to 0.716) | <0.001 |
| TyG_to_ArI_to_HADS_A_reverse | Total | 108 | 0.586 (0.389 to 0.756) | <0.001 |
| TyG_to_ArI_to_HADS_A_reverse | Proportion | 108 | 0.061 (-0.056 to 0.211) | 0.306 |
| TyG_to_ArI_to_HADS_A_reverse | a path | 108 | 0.323 (0.168 to 0.517) | <0.001 |
| TyG_to_ArI_to_HADS_A_reverse | b path | 108 | 0.111 (-0.090 to 0.296) | 0.306 |
| ArI_to_TyG_to_HADS_D | Indirect | 108 | 0.226 (0.116 to 0.376) | <0.001 |
| ArI_to_TyG_to_HADS_D | Direct | 108 | 0.117 (-0.045 to 0.272) | 0.147 |
| ArI_to_TyG_to_HADS_D | Total | 108 | 0.344 (0.167 to 0.542) | <0.001 |
| ArI_to_TyG_to_HADS_D | Proportion | 108 | 0.659 (0.386 to 1.218) | <0.001 |
| ArI_to_TyG_to_HADS_D | a path | 108 | 0.398 (0.232 to 0.596) | <0.001 |
| ArI_to_TyG_to_HADS_D | b path | 108 | 0.568 (0.353 to 0.749) | <0.001 |
| TyG_to_ArI_to_HADS_D_reverse | Indirect | 108 | 0.038 (-0.015 to 0.102) | 0.141 |
| TyG_to_ArI_to_HADS_D_reverse | Direct | 108 | 0.568 (0.349 to 0.749) | <0.001 |
| TyG_to_ArI_to_HADS_D_reverse | Total | 108 | 0.606 (0.391 to 0.798) | <0.001 |
| TyG_to_ArI_to_HADS_D_reverse | Proportion | 108 | 0.063 (-0.027 to 0.184) | 0.141 |
| TyG_to_ArI_to_HADS_D_reverse | a path | 108 | 0.323 (0.169 to 0.523) | <0.001 |
| TyG_to_ArI_to_HADS_D_reverse | b path | 108 | 0.117 (-0.043 to 0.272) | 0.141 |

Note: Reverse-order analyses demonstrate that variable ordering should not be interpreted causally.

**Part F. Exploratory sex-stratified analyses**

| **Sex** | **Outcome** | **n** | **Status** | **Predictor** | **Beta (95% CI)** | **P** |
| --- | --- | --- | --- | --- | --- | --- |
| Female | Education-corrected MoCA | 12 | Sparse subgroup |  |  |  |
| Female | HADS-A | 12 | Sparse subgroup |  |  |  |
| Female | HADS-D | 12 | Sparse subgroup |  |  |  |
| Male | Education-corrected MoCA | 96 | ok | AHI | -0.510 (-0.624 to -0.397) | <0.001 |
| Male | Education-corrected MoCA | 96 | ok | ArI | -0.106 (-0.231 to 0.019) | 0.094 |
| Male | Education-corrected MoCA | 96 | ok | TyG | -0.494 (-0.647 to -0.342) | <0.001 |
| Male | HADS-A | 96 | ok | AHI | 0.170 (-0.039 to 0.379) | 0.110 |
| Male | HADS-A | 96 | ok | ArI | 0.101 (-0.114 to 0.315) | 0.353 |
| Male | HADS-A | 96 | ok | TyG | 0.569 (0.352 to 0.786) | <0.001 |
| Male | HADS-D | 96 | ok | AHI | 0.201 (0.016 to 0.385) | 0.033 |
| Male | HADS-D | 96 | ok | ArI | 0.096 (-0.089 to 0.281) | 0.304 |
| Male | HADS-D | 96 | ok | TyG | 0.584 (0.374 to 0.794) | <0.001 |

Note: Female subgroup estimates were not fitted where the subgroup was too sparse.

**Supplementary Table S1B. Competing-exposure mutual-adjustment sensitivity analyses in the 108-patient deep-phenotyping OSA cohort**

**Part A. Associations after adjustment for the competing PSG exposure**

| **Association** | **Competing exposure additionally adjusted** | **Regression coefficient β (95% CI)** | **P value** | **N** |
| --- | --- | --- | --- | --- |
| AHI → education-corrected MoCA | ArI | −0.076 (−0.098 to −0.054) | <0.001 | 108 |
| ArI → HADS-A | AHI | 0.115 (0.039 to 0.191) | 0.003 | 108 |
| ArI → HADS-D | AHI | 0.131 (0.060 to 0.202) | <0.001 | 108 |
| ArI → TyG | AHI | 0.019 (0.011 to 0.027) | <0.001 | 108 |
| AHI → TyG | ArI | −0.0016 (−0.0041 to 0.0008) | 0.188 | 108 |

**Part B. Key competing-exposure-adjusted exploratory cross-sectional decomposition results**

| **Prespecified ordering** | **Competing exposure additionally adjusted** | **Statistical component** | **Estimate (95% bootstrap CI)** | **Bootstrap P** | **N** |
| --- | --- | --- | --- | --- | --- |
| AHI → TyG → education-corrected MoCA | ArI | Indirect statistical component | 0.241 (−0.090 to 0.656) | 0.154 | 108 |
| AHI → TyG → education-corrected MoCA | ArI | Direct statistical component | −2.307 (−2.751 to −1.849) | <0.001 | 108 |
| ArI → TyG → HADS-A | AHI | Indirect statistical component | 0.626 (0.305 to 1.031) | <0.001 | 108 |
| ArI → TyG → HADS-D | AHI | Indirect statistical component | 0.708 (0.356 to 1.152) | <0.001 | 108 |

**Note:** Part A reports unstandardized regression coefficients from sensitivity models that additionally adjusted for the competing PSG exposure; AHI and ArI are expressed in events/h. Part B reports the key exploratory cross-sectional associational-decomposition components explicitly used to assess the reviewer-requested competing-exposure sensitivity, based on 5,000 nonparametric bootstrap resamples. Decomposition estimates are expressed in outcome-score units for a 1-SD increase in the specified exposure. These analyses assess robustness to measured competing PSG exposure but do not establish temporal mediation or independent causal pathways. MoCA models additionally accounted for education.

**Abbreviations:** AHI, apnea-hypopnea index; ArI, arousal index; TyG, triglyceride-glucose index; MoCA, Montreal Cognitive Assessment; HADS-A/D, Hospital Anxiety and Depression Scale-Anxiety/Depression; CI, confidence interval; PSG, polysomnography; OSA, obstructive sleep apnea.

**Supplementary Table S2. Full-proteome population analyses and the 21 cross-outcome proteins**

**Part A. Full-proteome analysis audit**

| **Outcome** | **QC protein rows** | **Estimable proteins** | **Median model N** | **N range** | **FDR <0.05** | **Nominal P <0.05** |
| --- | --- | --- | --- | --- | --- | --- |
| Cognition | 2,923 | 2,922 | 14,412 | 4,607 to 16,522 | 886 | 1,109 |
| PHQ-2 | 2,923 | 2,922 | 13,630 | 4,246 to 16,196 | 152 | 562 |
| GAD-2 | 2,923 | 2,922 | 13,612 | 4,252 to 16,170 | 45 | 433 |

Note: The QC universe contained 2,923 protein rows. Individual outcome models can contain a non-estimable protein and protein-specific missingness; therefore per-protein model N varies.

**Part B. Complete association estimates for the 21 cross-outcome proteins**

| **Protein** | **Cognition N** | **Cognition beta (95% CI)** | **COG FDR** | **PHQ-2 N** | **PHQ-2 OR (95% CI)** | **PHQ-2 FDR** | **GAD-2 N** | **GAD-2 OR (95% CI)** | **GAD-2 FDR** | **Direction** |
| --- | --- | --- | --- | --- | --- | --- | --- | --- | --- | --- |
| ACE2 | 16,012 | -0.029 (-0.043 to -0.016) | <0.001 | 15,705 | 1.143 (1.059 to 1.234) | 0.019 | 15,684 | 1.166 (1.087 to 1.251) | 0.008 | 3/3 adverse |
| ADAMTSL2 | 14,392 | -0.018 (-0.032 to -0.004) | 0.035 | 13,625 | 1.149 (1.062 to 1.243) | 0.018 | 13,607 | 1.153 (1.073 to 1.238) | 0.019 | 3/3 adverse |
| AGRN | 16,387 | -0.037 (-0.049 to -0.024) | <0.001 | 16,084 | 1.135 (1.055 to 1.222) | 0.020 | 16,058 | 1.130 (1.057 to 1.208) | 0.032 | 3/3 adverse |
| ALDH3A1 | 16,269 | -0.030 (-0.043 to -0.018) | <0.001 | 15,937 | 1.148 (1.076 to 1.226) | 0.003 | 15,913 | 1.118 (1.052 to 1.189) | 0.032 | 3/3 adverse |
| ALPP | 16,239 | -0.043 (-0.055 to -0.031) | <0.001 | 15,902 | 1.232 (1.151 to 1.318) | <0.001 | 15,878 | 1.173 (1.103 to 1.248) | 0.001 | 3/3 adverse |
| CEACAM6 | 14,392 | -0.016 (-0.029 to -0.003) | 0.048 | 13,625 | 1.138 (1.063 to 1.219) | 0.009 | 13,607 | 1.120 (1.051 to 1.192) | 0.032 | 3/3 adverse |
| FABP1 | 16,287 | -0.060 (-0.073 to -0.047) | <0.001 | 16,007 | 1.186 (1.101 to 1.278) | 0.001 | 15,981 | 1.135 (1.061 to 1.214) | 0.031 | 3/3 adverse |
| FGFR2 | 16,378 | -0.023 (-0.036 to -0.010) | 0.002 | 16,068 | 1.169 (1.083 to 1.262) | 0.004 | 16,042 | 1.138 (1.062 to 1.221) | 0.032 | 3/3 adverse |
| GCHFR | 13,707 | -0.017 (-0.030 to -0.003) | 0.048 | 13,006 | 1.170 (1.090 to 1.256) | 0.001 | 12,990 | 1.126 (1.055 to 1.203) | 0.032 | 3/3 adverse |
| GDF15 | 16,522 | -0.083 (-0.097 to -0.069) | <0.001 | 16,196 | 1.194 (1.113 to 1.280) | <0.001 | 16,170 | 1.130 (1.057 to 1.208) | 0.032 | 3/3 adverse |
| MMP12 | 16,337 | -0.067 (-0.080 to -0.054) | <0.001 | 16,012 | 1.196 (1.109 to 1.290) | <0.001 | 15,984 | 1.132 (1.057 to 1.213) | 0.032 | 3/3 adverse |
| MSLN | 16,213 | -0.021 (-0.033 to -0.008) | 0.005 | 15,875 | 1.178 (1.098 to 1.263) | <0.001 | 15,851 | 1.121 (1.051 to 1.196) | 0.036 | 3/3 adverse |
| OCLN | 14,337 | -0.032 (-0.045 to -0.018) | <0.001 | 13,584 | 1.218 (1.126 to 1.316) | <0.001 | 13,567 | 1.168 (1.090 to 1.253) | 0.008 | 3/3 adverse |
| PIGR | 16,106 | -0.035 (-0.047 to -0.023) | <0.001 | 15,800 | 1.254 (1.169 to 1.345) | <0.001 | 15,775 | 1.165 (1.093 to 1.242) | 0.004 | 3/3 adverse |
| REG4 | 16,331 | -0.032 (-0.044 to -0.019) | <0.001 | 16,030 | 1.200 (1.115 to 1.291) | <0.001 | 16,004 | 1.122 (1.049 to 1.199) | 0.047 | 3/3 adverse |
| RNASE6 | 14,266 | -0.041 (-0.055 to -0.028) | <0.001 | 13,517 | 1.163 (1.084 to 1.246) | 0.002 | 13,500 | 1.123 (1.051 to 1.200) | 0.040 | 3/3 adverse |
| SMPD1 | 16,393 | -0.040 (-0.052 to -0.028) | <0.001 | 16,109 | 1.131 (1.052 to 1.216) | 0.022 | 16,081 | 1.126 (1.055 to 1.201) | 0.032 | 3/3 adverse |
| TFF1 | 16,264 | -0.105 (-0.117 to -0.092) | <0.001 | 15,927 | 1.172 (1.093 to 1.257) | 0.001 | 15,902 | 1.126 (1.056 to 1.201) | 0.032 | 3/3 adverse |
| TFF2 | 16,364 | -0.063 (-0.076 to -0.051) | <0.001 | 16,056 | 1.174 (1.094 to 1.260) | 0.001 | 16,030 | 1.131 (1.060 to 1.205) | 0.030 | 3/3 adverse |
| TNFSF13B | 16,474 | -0.035 (-0.047 to -0.022) | <0.001 | 16,170 | 1.166 (1.086 to 1.253) | 0.002 | 16,144 | 1.138 (1.067 to 1.214) | 0.019 | 3/3 adverse |
| TREM2 | 16,287 | -0.023 (-0.036 to -0.010) | 0.003 | 16,007 | 1.243 (1.153 to 1.340) | <0.001 | 15,981 | 1.135 (1.059 to 1.215) | 0.032 | 3/3 adverse |

Note: Cognition is reported as standardized beta; PHQ-2 and GAD-2 are reported as odds ratios. The 21 proteins were defined by BH-FDR <0.05 in all three full-proteome outcome analyses. This is a population-derived cross-outcome association set and should not be described as an OSA-specific signature.

**Supplementary Table S3. Strictly adjusted analyses and null-model evaluation of the 21 candidate proteins**

**Part A. Strictly adjusted protein-outcome models**

| **Protein** | **Outcome** | **n** | **Strict effect (95% CI)** | **Strict FDR** | **Attenuation, %** |
| --- | --- | --- | --- | --- | --- |
| ACE2 | Cognition | 5,042 | -0.023 (-0.045 to -0.000) | 0.199 | 23.1 |
| ACE2 | GAD-2 | 5,042 | 1.107 (0.966 to 1.270) | 0.711 | 33.6 |
| ACE2 | PHQ-2 | 5,042 | 1.050 (0.901 to 1.223) | 0.560 | 63.8 |
| ADAMTSL2 | Cognition | 4,482 | -0.001 (-0.023 to 0.020) | 0.936 | 91.9 |
| ADAMTSL2 | GAD-2 | 4,482 | 1.082 (0.941 to 1.244) | 0.829 | 44.3 |
| ADAMTSL2 | PHQ-2 | 4,482 | 1.068 (0.915 to 1.246) | 0.448 | 52.7 |
| AGRN | Cognition | 5,165 | -0.024 (-0.045 to -0.003) | 0.153 | 34.7 |
| AGRN | GAD-2 | 5,165 | 1.106 (0.972 to 1.259) | 0.711 | 17.4 |
| AGRN | PHQ-2 | 5,165 | 1.003 (0.866 to 1.162) | 0.966 | 97.5 |
| ALDH3A1 | Cognition | 5,114 | -0.014 (-0.033 to 0.006) | 0.313 | 55.1 |
| ALDH3A1 | GAD-2 | 5,114 | 1.093 (0.973 to 1.228) | 0.711 | 20.2 |
| ALDH3A1 | PHQ-2 | 5,114 | 1.136 (1.003 to 1.287) | 0.117 | 7.6 |
| ALPP | Cognition | 5,111 | -0.018 (-0.038 to 0.001) | 0.199 | 57.6 |
| ALPP | GAD-2 | 5,111 | 0.948 (0.837 to 1.072) | 0.829 | 66.2 |
| ALPP | PHQ-2 | 5,111 | 1.300 (1.137 to 1.485) | 0.002 | -25.8 |
| CEACAM6 | Cognition | 4,482 | -0.011 (-0.032 to 0.010) | 0.467 | 32.4 |
| CEACAM6 | GAD-2 | 4,482 | 0.952 (0.834 to 1.087) | 0.829 | 56.7 |
| CEACAM6 | PHQ-2 | 4,482 | 1.157 (1.004 to 1.333) | 0.117 | -12.3 |
| FABP1 | Cognition | 5,132 | -0.019 (-0.040 to 0.001) | 0.199 | 67.9 |
| FABP1 | GAD-2 | 5,132 | 1.029 (0.902 to 1.174) | 0.829 | 77.5 |
| FABP1 | PHQ-2 | 5,132 | 1.092 (0.940 to 1.269) | 0.294 | 48.2 |
| FGFR2 | Cognition | 5,157 | -0.019 (-0.040 to 0.002) | 0.199 | 19.3 |
| FGFR2 | GAD-2 | 5,157 | 1.019 (0.890 to 1.166) | 0.919 | 85.7 |
| FGFR2 | PHQ-2 | 5,157 | 1.191 (1.024 to 1.385) | 0.117 | -11.9 |
| GCHFR | Cognition | 4,257 | -0.001 (-0.023 to 0.021) | 0.936 | 94.6 |
| GCHFR | GAD-2 | 4,257 | 1.000 (0.865 to 1.157) | 0.996 | 99.7 |
| GCHFR | PHQ-2 | 4,257 | 1.146 (0.995 to 1.320) | 0.139 | 13.4 |
| GDF15 | Cognition | 5,201 | -0.037 (-0.060 to -0.014) | 0.016 | 55.2 |
| GDF15 | GAD-2 | 5,201 | 0.945 (0.816 to 1.094) | 0.829 | 53.5 |
| GDF15 | PHQ-2 | 5,201 | 1.181 (1.024 to 1.363) | 0.117 | 5.9 |
| MMP12 | Cognition | 5,145 | -0.020 (-0.041 to 0.000) | 0.199 | 69.4 |
| MMP12 | GAD-2 | 5,145 | 1.101 (0.963 to 1.259) | 0.711 | 22.6 |
| MMP12 | PHQ-2 | 5,145 | 1.114 (0.954 to 1.301) | 0.239 | 39.4 |
| MSLN | Cognition | 5,104 | -0.012 (-0.031 to 0.008) | 0.416 | 44.1 |
| MSLN | GAD-2 | 5,104 | 0.910 (0.796 to 1.041) | 0.711 | 17.6 |
| MSLN | PHQ-2 | 5,104 | 1.252 (1.088 to 1.441) | 0.018 | -37.7 |
| OCLN | Cognition | 4,464 | -0.004 (-0.026 to 0.018) | 0.903 | 87.8 |
| OCLN | GAD-2 | 4,464 | 1.060 (0.926 to 1.213) | 0.829 | 62.6 |
| OCLN | PHQ-2 | 4,464 | 1.095 (0.937 to 1.279) | 0.294 | 53.9 |
| PIGR | Cognition | 5,055 | -0.017 (-0.037 to 0.002) | 0.199 | 50.0 |
| PIGR | GAD-2 | 5,055 | 1.029 (0.908 to 1.165) | 0.829 | 81.5 |
| PIGR | PHQ-2 | 5,055 | 1.163 (1.012 to 1.336) | 0.117 | 33.2 |
| REG4 | Cognition | 5,145 | -0.005 (-0.025 to 0.016) | 0.868 | 85.8 |
| REG4 | GAD-2 | 5,145 | 1.043 (0.919 to 1.185) | 0.829 | 63.0 |
| REG4 | PHQ-2 | 5,145 | 1.093 (0.951 to 1.255) | 0.277 | 51.4 |
| RNASE6 | Cognition | 4,434 | -0.018 (-0.040 to 0.005) | 0.243 | 57.6 |
| RNASE6 | GAD-2 | 4,434 | 1.060 (0.926 to 1.213) | 0.829 | 50.1 |
| RNASE6 | PHQ-2 | 4,434 | 1.112 (0.967 to 1.279) | 0.218 | 29.3 |
| SMPD1 | Cognition | 5,159 | -0.015 (-0.035 to 0.004) | 0.243 | 61.2 |
| SMPD1 | GAD-2 | 5,159 | 1.029 (0.907 to 1.168) | 0.829 | 75.5 |
| SMPD1 | PHQ-2 | 5,159 | 1.134 (0.980 to 1.311) | 0.173 | -1.7 |
| TFF1 | Cognition | 5,111 | -0.048 (-0.068 to -0.028) | <0.001 | 54.0 |
| TFF1 | GAD-2 | 5,111 | 1.030 (0.908 to 1.169) | 0.829 | 75.0 |
| TFF1 | PHQ-2 | 5,111 | 1.117 (0.973 to 1.284) | 0.205 | 30.1 |
| TFF2 | Cognition | 5,157 | -0.002 (-0.022 to 0.018) | 0.936 | 97.3 |
| TFF2 | GAD-2 | 5,157 | 1.034 (0.910 to 1.175) | 0.829 | 72.7 |
| TFF2 | PHQ-2 | 5,157 | 1.129 (0.982 to 1.297) | 0.173 | 24.6 |
| TNFSF13B | Cognition | 5,188 | -0.009 (-0.029 to 0.012) | 0.556 | 74.7 |
| TNFSF13B | GAD-2 | 5,188 | 1.004 (0.879 to 1.147) | 0.996 | 96.8 |
| TNFSF13B | PHQ-2 | 5,188 | 1.116 (0.959 to 1.298) | 0.232 | 28.5 |
| TREM2 | Cognition | 5,132 | 0.002 (-0.019 to 0.023) | 0.936 | 92.3 |
| TREM2 | GAD-2 | 5,132 | 1.010 (0.883 to 1.155) | 0.981 | 92.4 |
| TREM2 | PHQ-2 | 5,132 | 1.171 (1.010 to 1.358) | 0.117 | 27.2 |

Note: Strict models incorporate prespecified mutual-outcome and inflammatory-burden adjustment.

**Part B. Strict-model summary**

| **Outcome** | **Direction retained** | **FDR significant** | **Nominal P <0.05** |
| --- | --- | --- | --- |
| Cognition | 20 | 2 | 4 |
| GAD-2 | 17 | 0 | 0 |
| PHQ-2 | 21 | 2 | 8 |

Note: FDR counts are reported separately for each outcome.

**Part C. Correlation-preserving permutation analysis**

| **Statistic** | **Observed** | **Null mean** | **Null 95% range** | **Empirical P** | **B** |
| --- | --- | --- | --- | --- | --- |
| 3-of-3 adverse directions | 21 | 3.929 | 0 to 11 | 0.00020 | 5,000 |
| At least 2-of-3 adverse directions | 21 | 10.538 | 3 to 18 | 0.002 | 5,000 |

Note: This is a post-selection descriptive sensitivity analysis and does not provide independent replication.

**Part D. Missingness-matched random-set analysis**

| **Statistic** | **Observed** | **Matched null mean** | **Matched null 95% range** | **Empirical P** | **B** |
| --- | --- | --- | --- | --- | --- |
| 3-of-3 adverse directions | 21 | 8.795 | 5 to 13 | 0.00010 | 10,000 |
| At least 2-of-3 adverse directions | 21 | 16.379 | 13 to 20 | 0.005 | 10,000 |

Note: Random protein sets were matched for missingness structure.

**Supplementary Table S4. Participant-level OSA-definition audit and denominator-aware contextual assessment of the 21 population-derived cross-outcome proteins**

**Part A. Participant-level OSA-definition and analysis-denominator audit**

| **OSA definition** | **Field-based definition / temporal rule** | **Primary BMI-adjusted TyG-WHtR analytic N (PHQ-2 ≥4 / <4)** | **Primary risk-model development N (PHQ-2 ≥4 / <4)** | **21-protein OSA-context outcome evaluability** |
| --- | --- | --- | --- | --- |
| Pre-MHQ available-source OSA | Baseline Data-Field 20002 code 1123 OR inpatient Data-Field 41270 G47.3 with the paired Data-Field 41280 diagnosis date on/before Data-Field 20400 MHQ completion | 619 (48/571) | 724 (63/661) | Cognition not estimable; PHQ-2/GAD-2 estimable |
| Ever-recorded available-source OSA | Any qualifying Data-Field 20002 code 1123 OR inpatient Data-Field 41270 G47.3 record; no temporal restriction | 894 (59/835) | Not used for primary risk-model development | All three outcomes estimable |
| Pre-MHQ ICD-defined OSA | Inpatient Data-Field 41270 G47.3 with the paired Data-Field 41280 diagnosis date on/before Data-Field 20400 MHQ completion | Not estimable | Not used for primary risk-model development | Cognition not estimable; PHQ-2/GAD-2 estimable |
| Ever-recorded ICD-defined OSA | Any inpatient Data-Field 41270 G47.3 record; no temporal restriction | 329 (12/317) | Not used for primary risk-model development | All three outcomes estimable |

**Note.** Counts in Part A are analysis-specific participant denominators after the outcome and covariates required by each analysis; they are not raw UK Biobank OSA prevalence counts. For the TyG-WHtR and risk-model columns, cases/controls denote PHQ-2 ≥4/<4 symptom-burden categories rather than OSA status. OSA-context proteomic models were restricted to OSA-positive participants under each definition. The primary risk model used the pre-MHQ available-source definition only.

**Part B. Denominator-aware candidate-set evaluability and directional-concordance summary**

| **OSA definition** | **Candidate proteins, total** | **Proteins with all 3 outcomes estimable** | **Three-outcome evaluability** | **3/3 directional concordance** | **≥2/3 directional concordance** | **Proteins with both MHQ outcomes estimable** | **2/2 PHQ-2/GAD-2 directional concordance** | **Proteins with ≥1 nominal P<0.05** | **Proteins with ≥1 BH-FDR<0.05** |
| --- | --- | --- | --- | --- | --- | --- | --- | --- | --- |
| Pre-MHQ available-source OSA | 21 | 0 | Not evaluable | Not evaluable | Not evaluable | 21 | 14/21 | 2 | 0 |
| Ever-recorded available-source OSA | 21 | 21 | Fully evaluable | 8/21 | 18/21 | 21 | 15/21 | 3 | 0 |
| Pre-MHQ ICD-defined OSA | 21 | 0 | Not evaluable | Not evaluable | Not evaluable | 21 | 16/21 | 3 | 0 |
| Ever-recorded ICD-defined OSA | 21 | 21 | Fully evaluable | 9/21 | 19/21 | 21 | 17/21 | 3 | 0 |

**Note.** Three-outcome directional concordance was calculated only when cognition, PHQ-2, and GAD-2 were all estimable. When cognition was not estimable, three-outcome concordance was reported as not evaluable, and concordance across the two MHQ outcomes (PHQ-2 and GAD-2) was reported separately. Nominal and FDR-supported associations refer to the corresponding OSA-context models.

**Abbreviations:** BH-FDR, Benjamini-Hochberg false discovery rate; GAD-2, Generalized Anxiety Disorder-2; MHQ, Mental Health Questionnaire; OSA, obstructive sleep apnea; PHQ-2, Patient Health Questionnaire-2.

**Supplementary Table S5. Complete audit of the 117 predefined machine-learning workflows**

**Part A. Workflow-count audit**

| **Item** | **Observed** | **Expected** | **Matches expected** |
| --- | --- | --- | --- |
| Selectors | 13 | 13 | true |
| Classifiers | 9 | 9 | true |
| Theoretical workflows | 117 | 117 | true |
| Successfully fitted workflows | 104 | 104 | true |
| Performance-evaluable workflows | 103 | 103 | true |
| Fitting failures | 13 | 13 | true |
| Post-fit non-evaluable workflows | 1 | 1 | true |
| Failed/non-evaluable in audit denominator | 14 | 14 | true |

Note: Fitting and evaluability counts are reported separately. Of 117 prespecified workflows, 104 produced fitted objects; 103 were performance-evaluable; 13 xgbTree workflows failed during fitting and one EN_0.8 + svmRadial workflow was post-fit non-evaluable. All 14 failed/non-evaluable workflows were retained in the audit denominator.

**Part B. Failed workflow summary**

| **Failure reason** | **Failed workflows, n** | **Workflow IDs** | **Selector + classifier pairs** |
| --- | --- | --- | --- |
| Stopping (13); no evaluable selection probabilities (1) | 14 | 2, 11, 20, 29, 38, 47, 56, 65, 74, 83, 92, 101, 110; 67 | EN_0.1 + xgbTree \| EN_0.2 + xgbTree \| EN_0.3 + xgbTree \| EN_0.4 + xgbTree \| EN_0.5 + xgbTree \| EN_0.6 + xgbTree \| EN_0.7 + xgbTree \| EN_0.8 + xgbTree \| EN_0.9 + xgbTree \| LASSO + xgbTree \| Ridge + xgbTree \| RF_importance + xgbTree \| XGB_gain + xgbTree \| EN_0.8 + svmRadial |

Note: All failed or non-evaluable workflows are retained transparently.

**Part C. Formal selection versus post-hoc best locked-validation workflow**

| **Selection rule** | **Workflow ID** | **Selector** | **Classifier** | **N features** | **Selection AUROC** | **Selection PR-AUC** | **Locked-validation AUROC** | **Locked-validation PR-AUC** | **Locked-validation Brier** |
| --- | --- | --- | --- | --- | --- | --- | --- | --- | --- |
| FORMAL: selection AUROC, then selection PR-AUC, parsimony, workflow ID | 25 | EN_0.3 | LDA | 148 | 0.696 | 0.443 | 0.694 | 0.438 | 0.172 |
| POST HOC ONLY: highest locked-validation AUROC; not used for formal selection | 77 | EN_0.9 | GBM | 171 | 0.688 | 0.425 | 0.698 | 0.431 | 0.170 |

Note: Formal workflow selection was based on the prespecified selection partition. The workflow with the highest locked-validation AUROC is shown only post hoc and was not used for formal model selection.

**Supplementary Table S5B. Complete row-level results for all 117 predefined machine-learning workflows**

| **workflow_id** | **selector** | **classifier** | **n_features** | **status** | **formal_status** | **formal_failure_reason** | **selection_AUROC** | **selection_PRAUC** | **selection_Brier** | **locked_validation_AUROC** | **locked_validation_PRAUC** | **locked_validation_Brier** |
| --- | --- | --- | --- | --- | --- | --- | --- | --- | --- | --- | --- | --- |
| 1 | EN_0.1 | GLM | 260 | Success | Success |  | 0.6876206 | 0.4211792 | 0.1754554 | 0.6873549 | 0.4373184 | 0.1739631 |
| 2 | EN_0.1 | xgbTree | 260 | Failed | Failed | Stopping |  |  |  |  |  |  |
| 3 | EN_0.1 | RF | 260 | Success | Success |  | 0.6648262 | 0.3984543 | 0.1768981 | 0.6760051 | 0.4042985 | 0.1762391 |
| 4 | EN_0.1 | svmRadial | 260 | Success | Success |  | 0.6754501 | 0.4342689 | 0.1722914 | 0.6868885 | 0.4431555 | 0.1710002 |
| 5 | EN_0.1 | GBM | 260 | Success | Success |  | 0.6817990 | 0.4206679 | 0.1726748 | 0.6906318 | 0.4255855 | 0.1716434 |
| 6 | EN_0.1 | glmboost | 260 | Success | Success |  | 0.6921170 | 0.4242720 | 0.1722275 | 0.6922691 | 0.4238033 | 0.1724098 |
| 7 | EN_0.1 | LDA | 260 | Success | Success |  | 0.6893127 | 0.4237794 | 0.1750989 | 0.6884993 | 0.4387629 | 0.1740835 |
| 8 | EN_0.1 | naive_bayes | 260 | Success | Success |  | 0.6494451 | 0.3772432 | 0.3058440 | 0.6647377 | 0.3822988 | 0.2946832 |
| 9 | EN_0.1 | plsRglm | 260 | Success | Success |  | 0.3504605 | 0.1868337 | 0.4708672 | 0.3448665 | 0.1841534 | 0.4692135 |
| 10 | EN_0.2 | GLM | 275 | Success | Success |  | 0.6881647 | 0.4247179 | 0.1756637 | 0.6855103 | 0.4324337 | 0.1756759 |
| 11 | EN_0.2 | xgbTree | 275 | Failed | Failed | Stopping |  |  |  |  |  |  |
| 12 | EN_0.2 | RF | 275 | Success | Success |  | 0.6617439 | 0.3935214 | 0.1776665 | 0.6711652 | 0.4070307 | 0.1767578 |
| 13 | EN_0.2 | svmRadial | 275 | Success | Success |  | 0.6805102 | 0.4357753 | 0.1720511 | 0.6890736 | 0.4450751 | 0.1707202 |
| 14 | EN_0.2 | GBM | 275 | Success | Success |  | 0.6864628 | 0.4233298 | 0.1715386 | 0.6940584 | 0.4363783 | 0.1703156 |
| 15 | EN_0.2 | glmboost | 275 | Success | Success |  | 0.6921170 | 0.4242720 | 0.1722275 | 0.6922691 | 0.4238033 | 0.1724098 |
| 16 | EN_0.2 | LDA | 275 | Success | Success |  | 0.6899259 | 0.4293653 | 0.1751984 | 0.6874781 | 0.4351609 | 0.1756337 |
| 17 | EN_0.2 | naive_bayes | 275 | Success | Success |  | 0.6461016 | 0.3747239 | 0.3024228 | 0.6633906 | 0.3801617 | 0.2913495 |
| 18 | EN_0.2 | plsRglm | 275 | Success | Success |  | 0.3518766 | 0.1871188 | 0.4701337 | 0.3463587 | 0.1848301 | 0.4687226 |
| 19 | EN_0.3 | GLM | 148 | Success | Success |  | 0.6956113 | 0.4422579 | 0.1706313 | 0.6918989 | 0.4360516 | 0.1718872 |
| 20 | EN_0.3 | xgbTree | 148 | Failed | Failed | Stopping |  |  |  |  |  |  |
| 21 | EN_0.3 | RF | 148 | Success | Success |  | 0.6649116 | 0.4051079 | 0.1761889 | 0.6851450 | 0.4169936 | 0.1745342 |
| 22 | EN_0.3 | svmRadial | 148 | Success | Success |  | 0.6612093 | 0.4360509 | 0.1731881 | 0.6768417 | 0.4354455 | 0.1731557 |
| 23 | EN_0.3 | GBM | 148 | Success | Success |  | 0.6852794 | 0.4252371 | 0.1716256 | 0.6949991 | 0.4339745 | 0.1704425 |
| 24 | EN_0.3 | glmboost | 148 | Success | Success |  | 0.6921170 | 0.4242720 | 0.1722275 | 0.6922691 | 0.4238033 | 0.1724098 |
| 25 | EN_0.3 | LDA | 148 | Success | Success |  | 0.6962823 | 0.4432656 | 0.1707487 | 0.6938682 | 0.4384156 | 0.1718690 |
| 26 | EN_0.3 | naive_bayes | 148 | Success | Success |  | 0.6606493 | 0.3895640 | 0.2746942 | 0.6732530 | 0.3901069 | 0.2695126 |
| 27 | EN_0.3 | plsRglm | 148 | Success | Success |  | 0.3405757 | 0.1837574 | 0.4749822 | 0.3329354 | 0.1813587 | 0.4736425 |
| 28 | EN_0.4 | GLM | 178 | Success | Success |  | 0.6920722 | 0.4296491 | 0.1729951 | 0.6897822 | 0.4387689 | 0.1724209 |
| 29 | EN_0.4 | xgbTree | 178 | Failed | Failed | Stopping |  |  |  |  |  |  |
| 30 | EN_0.4 | RF | 178 | Success | Success |  | 0.6630600 | 0.4049917 | 0.1767600 | 0.6847623 | 0.4221391 | 0.1748079 |
| 31 | EN_0.4 | svmRadial | 178 | Success | Success |  | 0.6746336 | 0.4393163 | 0.1720659 | 0.6818335 | 0.4408770 | 0.1721688 |
| 32 | EN_0.4 | GBM | 178 | Success | Success |  | 0.6847898 | 0.4202793 | 0.1722039 | 0.6884467 | 0.4299959 | 0.1714705 |
| 33 | EN_0.4 | glmboost | 178 | Success | Success |  | 0.6921170 | 0.4242720 | 0.1722275 | 0.6922691 | 0.4238033 | 0.1724098 |
| 34 | EN_0.4 | LDA | 178 | Success | Success |  | 0.6935494 | 0.4323186 | 0.1728661 | 0.6912800 | 0.4403490 | 0.1725405 |
| 35 | EN_0.4 | naive_bayes | 178 | Success | Success |  | 0.6561142 | 0.3843485 | 0.2827105 | 0.6708999 | 0.3867232 | 0.2741489 |
| 36 | EN_0.4 | plsRglm | 178 | Success | Success |  | 0.3447196 | 0.1848409 | 0.4734817 | 0.3365992 | 0.1823136 | 0.4721096 |
| 37 | EN_0.5 | GLM | 143 | Success | Success |  | 0.6951255 | 0.4399124 | 0.1710611 | 0.6911656 | 0.4363909 | 0.1719615 |
| 38 | EN_0.5 | xgbTree | 143 | Failed | Failed | Stopping |  |  |  |  |  |  |
| 39 | EN_0.5 | RF | 143 | Success | Success |  | 0.6678809 | 0.4158535 | 0.1755829 | 0.6858441 | 0.4206663 | 0.1744433 |
| 40 | EN_0.5 | svmRadial | 143 | Success | Success |  | 0.6617744 | 0.4353241 | 0.1732686 | 0.6753765 | 0.4350979 | 0.1731252 |
| 41 | EN_0.5 | GBM | 143 | Success | Success |  | 0.6846746 | 0.4236231 | 0.1720834 | 0.6871745 | 0.4246278 | 0.1720751 |
| 42 | EN_0.5 | glmboost | 143 | Success | Success |  | 0.6921170 | 0.4242720 | 0.1722275 | 0.6922691 | 0.4238033 | 0.1724098 |
| 43 | EN_0.5 | LDA | 143 | Success | Success |  | 0.6957512 | 0.4411537 | 0.1712278 | 0.6930712 | 0.4387692 | 0.1719701 |
| 44 | EN_0.5 | naive_bayes | 143 | Success | Success |  | 0.6609537 | 0.3888079 | 0.2728560 | 0.6718541 | 0.3881288 | 0.2691210 |
| 45 | EN_0.5 | plsRglm | 143 | Success | Success |  | 0.3398259 | 0.1834486 | 0.4751157 | 0.3331377 | 0.1812763 | 0.4737034 |
| 46 | EN_0.6 | GLM | 142 | Success | Success |  | 0.6944713 | 0.4395605 | 0.1711621 | 0.6901984 | 0.4364290 | 0.1720856 |
| 47 | EN_0.6 | xgbTree | 142 | Failed | Failed | Stopping |  |  |  |  |  |  |
| 48 | EN_0.6 | RF | 142 | Success | Success |  | 0.6676407 | 0.4112162 | 0.1758382 | 0.6801097 | 0.4161450 | 0.1748857 |
| 49 | EN_0.6 | svmRadial | 142 | Success | Success |  | 0.6612111 | 0.4347974 | 0.1734323 | 0.6753579 | 0.4354973 | 0.1731070 |
| 50 | EN_0.6 | GBM | 142 | Success | Success |  | 0.6871543 | 0.4234002 | 0.1717158 | 0.6936334 | 0.4298267 | 0.1709045 |
| 51 | EN_0.6 | glmboost | 142 | Success | Success |  | 0.6921170 | 0.4242720 | 0.1722275 | 0.6922691 | 0.4238033 | 0.1724098 |
| 52 | EN_0.6 | LDA | 142 | Success | Success |  | 0.6951693 | 0.4418260 | 0.1713050 | 0.6922719 | 0.4383034 | 0.1720999 |
| 53 | EN_0.6 | naive_bayes | 142 | Success | Success |  | 0.6609006 | 0.3892695 | 0.2727914 | 0.6718973 | 0.3878032 | 0.2691184 |
| 54 | EN_0.6 | plsRglm | 142 | Success | Success |  | 0.3398259 | 0.1834486 | 0.4751157 | 0.3331377 | 0.1812763 | 0.4737034 |
| 55 | EN_0.7 | GLM | 207 | Success | Success |  | 0.6908897 | 0.4235265 | 0.1743064 | 0.6854335 | 0.4364392 | 0.1741700 |
| 56 | EN_0.7 | xgbTree | 207 | Failed | Failed | Stopping |  |  |  |  |  |  |
| 57 | EN_0.7 | RF | 207 | Success | Success |  | 0.6605919 | 0.3931020 | 0.1775413 | 0.6826521 | 0.4170876 | 0.1755805 |
| 58 | EN_0.7 | svmRadial | 207 | Success | Success |  | 0.6781391 | 0.4378712 | 0.1719180 | 0.6809821 | 0.4411714 | 0.1719369 |
| 59 | EN_0.7 | GBM | 207 | Success | Success |  | 0.6868825 | 0.4326466 | 0.1709316 | 0.6898887 | 0.4271536 | 0.1716575 |
| 60 | EN_0.7 | glmboost | 207 | Success | Success |  | 0.6919841 | 0.4245093 | 0.1722510 | 0.6917896 | 0.4228196 | 0.1725104 |
| 61 | EN_0.7 | LDA | 207 | Success | Success |  | 0.6918083 | 0.4274590 | 0.1740386 | 0.6871954 | 0.4375296 | 0.1742990 |
| 62 | EN_0.7 | naive_bayes | 207 | Success | Success |  | 0.6579506 | 0.3851033 | 0.2877738 | 0.6689571 | 0.3849829 | 0.2796010 |
| 63 | EN_0.7 | plsRglm | 207 | Success | Success |  | 0.3436737 | 0.1843941 | 0.4738559 | 0.3386410 | 0.1826679 | 0.4721856 |
| 64 | EN_0.8 | GLM | 206 | Success | Success |  | 0.6907055 | 0.4241331 | 0.1743131 | 0.6843059 | 0.4359276 | 0.1744321 |
| 65 | EN_0.8 | xgbTree | 206 | Failed | Failed | Stopping |  |  |  |  |  |  |
| 66 | EN_0.8 | RF | 206 | Success | Success |  | 0.6685034 | 0.3974371 | 0.1768624 | 0.6762672 | 0.4082602 | 0.1761943 |
| 67 | EN_0.8 | svmRadial | 206 | Non-evaluable | Non-evaluable | No evaluable selection probabilities |  |  |  |  |  |  |
| 68 | EN_0.8 | GBM | 206 | Success | Success |  | 0.6848495 | 0.4244619 | 0.1718017 | 0.6905304 | 0.4234513 | 0.1717686 |
| 69 | EN_0.8 | glmboost | 206 | Success | Success |  | 0.6919841 | 0.4245093 | 0.1722510 | 0.6917896 | 0.4228196 | 0.1725104 |
| 70 | EN_0.8 | LDA | 206 | Success | Success |  | 0.6915588 | 0.4272112 | 0.1740372 | 0.6858795 | 0.4373855 | 0.1745540 |
| 71 | EN_0.8 | naive_bayes | 206 | Success | Success |  | 0.6580884 | 0.3851545 | 0.2879271 | 0.6686070 | 0.3846870 | 0.2797965 |
| 72 | EN_0.8 | plsRglm | 206 | Success | Success |  | 0.3437357 | 0.1843717 | 0.4737944 | 0.3386238 | 0.1826762 | 0.4721222 |
| 73 | EN_0.9 | GLM | 171 | Success | Success |  | 0.6923058 | 0.4316871 | 0.1727352 | 0.6899575 | 0.4415297 | 0.1721322 |
| 74 | EN_0.9 | xgbTree | 171 | Failed | Failed | Stopping |  |  |  |  |  |  |
| 75 | EN_0.9 | RF | 171 | Success | Success |  | 0.6685752 | 0.4068457 | 0.1761753 | 0.6829135 | 0.4108187 | 0.1751743 |
| 76 | EN_0.9 | svmRadial | 171 | Success | Success |  | 0.6738489 | 0.4389123 | 0.1721116 | 0.6804334 | 0.4371843 | 0.1725556 |
| 77 | EN_0.9 | GBM | 171 | Success | Success |  | 0.6876784 | 0.4245646 | 0.1714976 | 0.6977175 | 0.4310828 | 0.1702924 |
| 78 | EN_0.9 | glmboost | 171 | Success | Success |  | 0.6919841 | 0.4245093 | 0.1722510 | 0.6917896 | 0.4228196 | 0.1725104 |
| 79 | EN_0.9 | LDA | 171 | Success | Success |  | 0.6934142 | 0.4333891 | 0.1726918 | 0.6914865 | 0.4428473 | 0.1723116 |
| 80 | EN_0.9 | naive_bayes | 171 | Success | Success |  | 0.6565492 | 0.3844570 | 0.2793384 | 0.6705674 | 0.3863504 | 0.2718261 |
| 81 | EN_0.9 | plsRglm | 171 | Success | Success |  | 0.3419265 | 0.1839396 | 0.4743665 | 0.3351875 | 0.1817992 | 0.4729653 |
| 82 | LASSO | GLM | 172 | Success | Success |  | 0.6924774 | 0.4325823 | 0.1726106 | 0.6898869 | 0.4419539 | 0.1720851 |
| 83 | LASSO | xgbTree | 172 | Failed | Failed | Stopping |  |  |  |  |  |  |
| 84 | LASSO | RF | 172 | Success | Success |  | 0.6672544 | 0.4055272 | 0.1763558 | 0.6801748 | 0.4150244 | 0.1754379 |
| 85 | LASSO | svmRadial | 172 | Success | Success |  | 0.6743865 | 0.4392089 | 0.1720296 | 0.6803776 | 0.4376211 | 0.1724328 |
| 86 | LASSO | GBM | 172 | Success | Success |  | 0.6826579 | 0.4273726 | 0.1719765 | 0.6925820 | 0.4269793 | 0.1713646 |
| 87 | LASSO | glmboost | 172 | Success | Success |  | 0.6919841 | 0.4245093 | 0.1722510 | 0.6917896 | 0.4228196 | 0.1725104 |
| 88 | LASSO | LDA | 172 | Success | Success |  | 0.6936389 | 0.4343162 | 0.1725654 | 0.6914976 | 0.4433328 | 0.1722592 |
| 89 | LASSO | naive_bayes | 172 | Success | Success |  | 0.6564802 | 0.3848126 | 0.2794818 | 0.6705311 | 0.3863366 | 0.2720527 |
| 90 | LASSO | plsRglm | 172 | Success | Success |  | 0.3419265 | 0.1839396 | 0.4743665 | 0.3351875 | 0.1817992 | 0.4729653 |
| 91 | Ridge | GLM | 30 | Success | Success |  | 0.6865943 | 0.4166528 | 0.1721330 | 0.6788458 | 0.4121637 | 0.1737935 |
| 92 | Ridge | xgbTree | 30 | Failed | Failed | Stopping |  |  |  |  |  |  |
| 93 | Ridge | RF | 30 | Success | Success |  | 0.6645875 | 0.3990780 | 0.1754831 | 0.6708369 | 0.3974619 | 0.1753079 |
| 94 | Ridge | svmRadial | 30 | Success | Success |  | 0.6387059 | 0.3970198 | 0.1778694 | 0.6381986 | 0.3918664 | 0.1792307 |
| 95 | Ridge | GBM | 30 | Success | Success |  | 0.6777004 | 0.4090997 | 0.1735925 | 0.6754378 | 0.4007935 | 0.1747229 |
| 96 | Ridge | glmboost | 30 | Success | Success |  | 0.6846368 | 0.4109328 | 0.1732415 | 0.6816982 | 0.4109920 | 0.1738064 |
| 97 | Ridge | LDA | 30 | Success | Success |  | 0.6870443 | 0.4180666 | 0.1721818 | 0.6813197 | 0.4143315 | 0.1736556 |
| 98 | Ridge | naive_bayes | 30 | Success | Success |  | 0.6555649 | 0.3773742 | 0.2278477 | 0.6692412 | 0.3866209 | 0.2217973 |
| 99 | Ridge | plsRglm | 30 | Success | Success |  | 0.3288565 | 0.1796522 | 0.4789693 | 0.3263659 | 0.1794094 | 0.4776720 |
| 100 | RF_importance | GLM | 30 | Success | Success |  | 0.6715706 | 0.4023958 | 0.1742356 | 0.6762832 | 0.4026206 | 0.1744480 |
| 101 | RF_importance | xgbTree | 30 | Failed | Failed | Stopping |  |  |  |  |  |  |
| 102 | RF_importance | RF | 30 | Success | Success |  | 0.6584447 | 0.3837298 | 0.1765500 | 0.6758884 | 0.4134322 | 0.1740356 |
| 103 | RF_importance | svmRadial | 30 | Success | Success |  | 0.6325905 | 0.3882281 | 0.1790762 | 0.6231888 | 0.3816111 | 0.1798617 |
| 104 | RF_importance | GBM | 30 | Success | Success |  | 0.6686106 | 0.3977071 | 0.1749233 | 0.6776310 | 0.4042650 | 0.1739181 |
| 105 | RF_importance | glmboost | 30 | Success | Success |  | 0.6691179 | 0.3974102 | 0.1748614 | 0.6763120 | 0.4018029 | 0.1745472 |
| 106 | RF_importance | LDA | 30 | Success | Success |  | 0.6714559 | 0.4031572 | 0.1744137 | 0.6768222 | 0.4045160 | 0.1744846 |
| 107 | RF_importance | naive_bayes | 30 | Success | Success |  | 0.6613837 | 0.3875729 | 0.2464194 | 0.6715639 | 0.3898485 | 0.2453061 |
| 108 | RF_importance | plsRglm | 30 | Success | Success |  | 0.3502035 | 0.1861581 | 0.4667192 | 0.3410046 | 0.1831280 | 0.4651350 |
| 109 | XGB_gain | GLM | 30 | Success | Success |  | 0.6779965 | 0.4104962 | 0.1733337 | 0.6812527 | 0.4146408 | 0.1728776 |
| 110 | XGB_gain | xgbTree | 30 | Failed | Failed | Stopping |  |  |  |  |  |  |
| 111 | XGB_gain | RF | 30 | Success | Success |  | 0.6592012 | 0.3920857 | 0.1761991 | 0.6770342 | 0.4110794 | 0.1739649 |
| 112 | XGB_gain | svmRadial | 30 | Success | Success |  | 0.6351057 | 0.3949353 | 0.1793908 | 0.6408719 | 0.4016262 | 0.1787975 |
| 113 | XGB_gain | GBM | 30 | Success | Success |  | 0.6735894 | 0.4067966 | 0.1744122 | 0.6742372 | 0.4052399 | 0.1746734 |
| 114 | XGB_gain | glmboost | 30 | Success | Success |  | 0.6778967 | 0.4080716 | 0.1737062 | 0.6820353 | 0.4130402 | 0.1733297 |
| 115 | XGB_gain | LDA | 30 | Success | Success |  | 0.6781499 | 0.4110572 | 0.1734739 | 0.6823255 | 0.4168858 | 0.1728452 |
| 116 | XGB_gain | naive_bayes | 30 | Success | Success |  | 0.6547074 | 0.3805137 | 0.2321329 | 0.6663619 | 0.3817065 | 0.2261830 |
| 117 | XGB_gain | plsRglm | 30 | Success | Success |  | 0.3420468 | 0.1837167 | 0.4705219 | 0.3293796 | 0.1813318 | 0.4715046 |

Note: All successful, failed, and non-evaluable workflows are retained. Locked-validation performance must not be used retrospectively to redefine the formally selected workflow.

**Supplementary Table S6. Protein feature-selection frequency and stability across the 117-workflow analysis**

| **Protein** | **Selectors, n/13** | **Selector frequency** | **Successful workflows containing feature** | **Successful-workflow frequency** | **Top-10 workflow frequency** | **Median selection AUROC** | **Maximum locked-validation AUROC** | **Selected in formal workflow** |
| --- | --- | --- | --- | --- | --- | --- | --- | --- |
| CDCP1 | 13 | 1.000 | 103 | 1.000 | 1.000 | 0.675 | 0.698 | Yes |
| TFF1 | 13 | 1.000 | 103 | 1.000 | 1.000 | 0.675 | 0.698 | Yes |
| GDF15 | 13 | 1.000 | 103 | 1.000 | 1.000 | 0.675 | 0.698 | Yes |
| GAST | 13 | 1.000 | 103 | 1.000 | 1.000 | 0.675 | 0.698 | Yes |
| SPINK5 | 13 | 1.000 | 103 | 1.000 | 1.000 | 0.675 | 0.698 | Yes |
| GHRL | 13 | 1.000 | 103 | 1.000 | 1.000 | 0.675 | 0.698 | Yes |
| REG3A | 13 | 1.000 | 103 | 1.000 | 1.000 | 0.675 | 0.698 | Yes |
| CA14 | 13 | 1.000 | 103 | 1.000 | 1.000 | 0.675 | 0.698 | Yes |
| CEACAM1 | 13 | 1.000 | 103 | 1.000 | 1.000 | 0.675 | 0.698 | Yes |
| ART3 | 13 | 1.000 | 103 | 1.000 | 1.000 | 0.675 | 0.698 | Yes |
| EDA2R | 12 | 0.923 | 96 | 0.932 | 1.000 | 0.675 | 0.698 | Yes |
| NOTCH2 | 12 | 0.923 | 96 | 0.932 | 1.000 | 0.675 | 0.698 | Yes |
| NEFL | 12 | 0.923 | 96 | 0.932 | 1.000 | 0.678 | 0.698 | Yes |
| CGA | 12 | 0.923 | 96 | 0.932 | 1.000 | 0.675 | 0.698 | Yes |
| THBS2 | 12 | 0.923 | 96 | 0.932 | 1.000 | 0.678 | 0.698 | Yes |
| L1CAM | 12 | 0.923 | 96 | 0.932 | 1.000 | 0.678 | 0.698 | Yes |
| IGFBP3 | 12 | 0.923 | 96 | 0.932 | 1.000 | 0.678 | 0.698 | Yes |
| SERPINA12 | 12 | 0.923 | 96 | 0.932 | 1.000 | 0.675 | 0.698 | Yes |
| MEPE | 12 | 0.923 | 96 | 0.932 | 1.000 | 0.678 | 0.698 | Yes |
| ADAMTS13 | 11 | 0.846 | 88 | 0.854 | 1.000 | 0.679 | 0.698 | Yes |
| ANGPTL1 | 11 | 0.846 | 88 | 0.854 | 1.000 | 0.679 | 0.698 | Yes |
| BST2 | 11 | 0.846 | 88 | 0.854 | 1.000 | 0.679 | 0.698 | Yes |
| CCL7 | 11 | 0.846 | 88 | 0.854 | 1.000 | 0.678 | 0.698 | Yes |
| CRTAC1 | 11 | 0.846 | 88 | 0.854 | 1.000 | 0.679 | 0.698 | Yes |
| CREB3 | 11 | 0.846 | 88 | 0.854 | 1.000 | 0.679 | 0.698 | Yes |
| MMP12 | 11 | 0.846 | 88 | 0.854 | 1.000 | 0.675 | 0.698 | Yes |
| FASLG | 11 | 0.846 | 88 | 0.854 | 1.000 | 0.679 | 0.698 | Yes |
| LTA | 11 | 0.846 | 88 | 0.854 | 1.000 | 0.679 | 0.698 | Yes |
| CD300LF | 11 | 0.846 | 88 | 0.854 | 1.000 | 0.678 | 0.698 | Yes |
| PAEP | 11 | 0.846 | 88 | 0.854 | 1.000 | 0.679 | 0.698 | Yes |
| SFRP1 | 11 | 0.846 | 88 | 0.854 | 1.000 | 0.675 | 0.698 | Yes |
| CCL24 | 11 | 0.846 | 88 | 0.854 | 1.000 | 0.679 | 0.698 | Yes |
| HMCN2 | 11 | 0.846 | 88 | 0.854 | 1.000 | 0.679 | 0.698 | Yes |
| STC2 | 11 | 0.846 | 88 | 0.854 | 1.000 | 0.678 | 0.698 | Yes |
| IL18 | 11 | 0.846 | 88 | 0.854 | 1.000 | 0.679 | 0.698 | Yes |
| KIR3DL2 | 11 | 0.846 | 88 | 0.854 | 1.000 | 0.679 | 0.698 | Yes |
| THRAP3 | 11 | 0.846 | 88 | 0.854 | 1.000 | 0.679 | 0.698 | Yes |
| DCBLD2 | 11 | 0.846 | 88 | 0.854 | 1.000 | 0.678 | 0.698 | Yes |
| PDXDC1 | 11 | 0.846 | 88 | 0.854 | 1.000 | 0.679 | 0.698 | Yes |
| ACP5 | 10 | 0.769 | 80 | 0.777 | 1.000 | 0.681 | 0.698 | Yes |
| ADAM12 | 10 | 0.769 | 80 | 0.777 | 1.000 | 0.681 | 0.698 | Yes |
| AFAP1 | 10 | 0.769 | 80 | 0.777 | 1.000 | 0.681 | 0.698 | Yes |
| AHNAK2 | 10 | 0.769 | 80 | 0.777 | 1.000 | 0.681 | 0.698 | Yes |
| ANGPTL2 | 10 | 0.769 | 80 | 0.777 | 1.000 | 0.681 | 0.698 | Yes |
| ARID4B | 10 | 0.769 | 80 | 0.777 | 1.000 | 0.681 | 0.698 | Yes |
| ATF2 | 10 | 0.769 | 80 | 0.777 | 1.000 | 0.681 | 0.698 | Yes |
| ATP6AP2 | 10 | 0.769 | 80 | 0.777 | 1.000 | 0.681 | 0.698 | Yes |
| BGN | 10 | 0.769 | 80 | 0.777 | 1.000 | 0.681 | 0.698 | Yes |
| C1QL2 | 10 | 0.769 | 80 | 0.777 | 1.000 | 0.681 | 0.698 | Yes |
| C4BPB | 10 | 0.769 | 80 | 0.777 | 1.000 | 0.681 | 0.698 | Yes |
| CA3 | 10 | 0.769 | 80 | 0.777 | 1.000 | 0.681 | 0.698 | Yes |
| CALB1 | 10 | 0.769 | 80 | 0.777 | 1.000 | 0.681 | 0.698 | Yes |
| CBLIF | 10 | 0.769 | 80 | 0.777 | 1.000 | 0.681 | 0.698 | Yes |
| CCL18 | 10 | 0.769 | 80 | 0.777 | 1.000 | 0.681 | 0.698 | Yes |
| CCL2 | 10 | 0.769 | 80 | 0.777 | 1.000 | 0.681 | 0.698 | Yes |
| CCL28 | 10 | 0.769 | 80 | 0.777 | 1.000 | 0.681 | 0.698 | Yes |
| CCL3 | 10 | 0.769 | 80 | 0.777 | 1.000 | 0.681 | 0.698 | Yes |
| CD177 | 10 | 0.769 | 80 | 0.777 | 1.000 | 0.681 | 0.698 | Yes |
| CD27 | 10 | 0.769 | 80 | 0.777 | 1.000 | 0.681 | 0.698 | Yes |
| CD300A | 10 | 0.769 | 80 | 0.777 | 1.000 | 0.681 | 0.698 | Yes |
| CD48 | 10 | 0.769 | 80 | 0.777 | 1.000 | 0.681 | 0.698 | Yes |
| CD7 | 10 | 0.769 | 80 | 0.777 | 1.000 | 0.681 | 0.698 | Yes |
| CNTN1 | 10 | 0.769 | 80 | 0.777 | 1.000 | 0.681 | 0.698 | Yes |
| CNTN3 | 10 | 0.769 | 80 | 0.777 | 1.000 | 0.681 | 0.698 | Yes |
| COL2A1 | 10 | 0.769 | 80 | 0.777 | 1.000 | 0.681 | 0.698 | Yes |
| COLEC12 | 10 | 0.769 | 80 | 0.777 | 1.000 | 0.681 | 0.698 | Yes |
| CPA4 | 10 | 0.769 | 80 | 0.777 | 1.000 | 0.681 | 0.698 | Yes |
| CPB2 | 10 | 0.769 | 80 | 0.777 | 1.000 | 0.681 | 0.698 | Yes |
| CRIM1 | 10 | 0.769 | 80 | 0.777 | 1.000 | 0.681 | 0.698 | Yes |
| CRLF1 | 10 | 0.769 | 80 | 0.777 | 1.000 | 0.681 | 0.698 | Yes |
| CRYBB2 | 10 | 0.769 | 80 | 0.777 | 1.000 | 0.681 | 0.698 | Yes |
| CTRL | 10 | 0.769 | 80 | 0.777 | 1.000 | 0.681 | 0.698 | Yes |
| DAPK2 | 10 | 0.769 | 80 | 0.777 | 1.000 | 0.681 | 0.698 | Yes |
| DCC | 10 | 0.769 | 80 | 0.777 | 1.000 | 0.681 | 0.698 | Yes |
| DEFB103A_DEFB103B | 10 | 0.769 | 80 | 0.777 | 1.000 | 0.681 | 0.698 | Yes |
| DKK3 | 10 | 0.769 | 80 | 0.777 | 1.000 | 0.681 | 0.698 | Yes |
| EFEMP1 | 10 | 0.769 | 80 | 0.777 | 1.000 | 0.681 | 0.698 | Yes |
| EGFLAM | 10 | 0.769 | 80 | 0.777 | 1.000 | 0.681 | 0.698 | Yes |
| EIF5A | 10 | 0.769 | 80 | 0.777 | 1.000 | 0.681 | 0.698 | Yes |
| ERVV_1 | 10 | 0.769 | 80 | 0.777 | 1.000 | 0.681 | 0.698 | Yes |
| FUT3_FUT5 | 10 | 0.769 | 80 | 0.777 | 1.000 | 0.681 | 0.698 | Yes |
| GALNT2 | 10 | 0.769 | 80 | 0.777 | 1.000 | 0.681 | 0.698 | Yes |
| GFAP | 10 | 0.769 | 80 | 0.777 | 1.000 | 0.681 | 0.698 | Yes |
| GGH | 10 | 0.769 | 80 | 0.777 | 1.000 | 0.681 | 0.698 | Yes |
| GNLY | 10 | 0.769 | 80 | 0.777 | 1.000 | 0.681 | 0.698 | Yes |
| GPR15L | 10 | 0.769 | 80 | 0.777 | 1.000 | 0.681 | 0.698 | Yes |
| HMMR | 10 | 0.769 | 80 | 0.777 | 1.000 | 0.681 | 0.698 | Yes |
| HRAS | 10 | 0.769 | 80 | 0.777 | 1.000 | 0.681 | 0.698 | Yes |
| HS6ST1 | 10 | 0.769 | 80 | 0.777 | 1.000 | 0.681 | 0.698 | Yes |
| IL1RAP | 10 | 0.769 | 80 | 0.777 | 1.000 | 0.681 | 0.698 | Yes |
| IL31RA | 10 | 0.769 | 80 | 0.777 | 1.000 | 0.681 | 0.698 | Yes |
| ITIH3 | 10 | 0.769 | 80 | 0.777 | 1.000 | 0.681 | 0.698 | Yes |
| KRT5 | 10 | 0.769 | 80 | 0.777 | 1.000 | 0.681 | 0.698 | Yes |
| LECT2 | 10 | 0.769 | 80 | 0.777 | 1.000 | 0.681 | 0.698 | Yes |
| LILRA3 | 10 | 0.769 | 80 | 0.777 | 1.000 | 0.681 | 0.698 | Yes |
| LPA | 10 | 0.769 | 80 | 0.777 | 1.000 | 0.681 | 0.698 | Yes |
| LY6D | 10 | 0.769 | 80 | 0.777 | 1.000 | 0.681 | 0.698 | Yes |
| MARCO | 10 | 0.769 | 80 | 0.777 | 1.000 | 0.681 | 0.698 | Yes |
| MATN3 | 10 | 0.769 | 80 | 0.777 | 1.000 | 0.681 | 0.698 | Yes |
| MFAP3L | 10 | 0.769 | 80 | 0.777 | 1.000 | 0.681 | 0.698 | Yes |
| MME | 10 | 0.769 | 80 | 0.777 | 1.000 | 0.681 | 0.698 | Yes |
| NCLN | 10 | 0.769 | 80 | 0.777 | 1.000 | 0.681 | 0.698 | Yes |
| NCR1 | 10 | 0.769 | 80 | 0.777 | 1.000 | 0.681 | 0.698 | Yes |
| NELL1 | 10 | 0.769 | 80 | 0.777 | 1.000 | 0.681 | 0.698 | Yes |
| NOS3 | 10 | 0.769 | 80 | 0.777 | 1.000 | 0.681 | 0.698 | Yes |
| NTPROBNP | 10 | 0.769 | 80 | 0.777 | 1.000 | 0.681 | 0.698 | Yes |
| NTRK2 | 10 | 0.769 | 80 | 0.777 | 1.000 | 0.681 | 0.698 | Yes |
| OGN | 10 | 0.769 | 80 | 0.777 | 1.000 | 0.681 | 0.698 | Yes |
| OSMR | 10 | 0.769 | 80 | 0.777 | 1.000 | 0.681 | 0.698 | Yes |
| PALLD | 10 | 0.769 | 80 | 0.777 | 1.000 | 0.681 | 0.698 | Yes |
| PCDH9 | 10 | 0.769 | 80 | 0.777 | 1.000 | 0.681 | 0.698 | Yes |
| POMC | 10 | 0.769 | 80 | 0.777 | 1.000 | 0.681 | 0.698 | Yes |
| PRG3 | 10 | 0.769 | 80 | 0.777 | 1.000 | 0.681 | 0.698 | Yes |
| PRR4 | 10 | 0.769 | 80 | 0.777 | 1.000 | 0.681 | 0.698 | Yes |
| PTH | 10 | 0.769 | 80 | 0.777 | 1.000 | 0.681 | 0.698 | Yes |
| PTPRF | 10 | 0.769 | 80 | 0.777 | 1.000 | 0.681 | 0.698 | Yes |
| PTPRZ1 | 10 | 0.769 | 80 | 0.777 | 1.000 | 0.681 | 0.698 | Yes |
| PZP | 10 | 0.769 | 80 | 0.777 | 1.000 | 0.681 | 0.698 | Yes |
| QPCT | 10 | 0.769 | 80 | 0.777 | 1.000 | 0.681 | 0.698 | Yes |
| RABEPK | 10 | 0.769 | 80 | 0.777 | 1.000 | 0.681 | 0.698 | Yes |
| RAP1A | 10 | 0.769 | 80 | 0.777 | 1.000 | 0.681 | 0.698 | Yes |
| RSPO3 | 10 | 0.769 | 80 | 0.777 | 1.000 | 0.681 | 0.698 | Yes |
| RTN4R | 10 | 0.769 | 80 | 0.777 | 1.000 | 0.681 | 0.698 | Yes |
| SCARF2 | 10 | 0.769 | 80 | 0.777 | 1.000 | 0.681 | 0.698 | Yes |
| SCG2 | 10 | 0.769 | 80 | 0.777 | 1.000 | 0.681 | 0.698 | Yes |
| SELPLG | 10 | 0.769 | 80 | 0.777 | 1.000 | 0.681 | 0.698 | Yes |
| SEMA3F | 10 | 0.769 | 80 | 0.777 | 1.000 | 0.681 | 0.698 | Yes |
| SERPINA11 | 10 | 0.769 | 80 | 0.777 | 1.000 | 0.681 | 0.698 | Yes |
| SHD | 10 | 0.769 | 80 | 0.777 | 1.000 | 0.681 | 0.698 | Yes |
| SLC16A1 | 10 | 0.769 | 80 | 0.777 | 1.000 | 0.681 | 0.698 | Yes |
| SLC34A3 | 10 | 0.769 | 80 | 0.777 | 1.000 | 0.681 | 0.698 | Yes |
| SMPD1 | 10 | 0.769 | 80 | 0.777 | 1.000 | 0.681 | 0.698 | Yes |
| SPINK2 | 10 | 0.769 | 80 | 0.777 | 1.000 | 0.681 | 0.698 | Yes |
| TFRC | 10 | 0.769 | 80 | 0.777 | 1.000 | 0.681 | 0.698 | Yes |
| TNFRSF11A | 10 | 0.769 | 80 | 0.777 | 1.000 | 0.681 | 0.698 | Yes |
| TNFSF12 | 10 | 0.769 | 80 | 0.777 | 1.000 | 0.681 | 0.698 | Yes |
| TRIM24 | 10 | 0.769 | 80 | 0.777 | 1.000 | 0.681 | 0.698 | Yes |
| TSPAN7 | 10 | 0.769 | 80 | 0.777 | 1.000 | 0.681 | 0.698 | Yes |
| VWA1 | 10 | 0.769 | 80 | 0.777 | 1.000 | 0.681 | 0.698 | Yes |
| COL15A1 | 9 | 0.692 | 72 | 0.699 | 0.800 | 0.681 | 0.698 | Yes |
| IL7R | 8 | 0.615 | 64 | 0.621 | 0.600 | 0.681 | 0.698 | Yes |
| MEP1A | 8 | 0.615 | 64 | 0.621 | 0.600 | 0.681 | 0.698 | Yes |
| TNFRSF10C | 8 | 0.615 | 64 | 0.621 | 0.600 | 0.681 | 0.698 | Yes |
| SEZ6L | 7 | 0.538 | 56 | 0.544 | 0.700 | 0.677 | 0.695 | Yes |
| CNTN2 | 6 | 0.462 | 48 | 0.466 | 0.300 | 0.681 | 0.695 | Yes |
| COL3A1 | 6 | 0.462 | 48 | 0.466 | 0.300 | 0.681 | 0.695 | Yes |
| PKD1 | 5 | 0.385 | 40 | 0.388 | 0.300 | 0.677 | 0.695 | Yes |
| TTR | 5 | 0.385 | 40 | 0.388 | 0.700 | 0.679 | 0.695 | Yes |

Note: Feature-selection frequency is used for candidate prioritization and robustness assessment. It is not evidence of causal relevance or an independently validated biomarker.

**Supplementary Table S7. TyG-WHtR association analyses across OSA definitions and label strategies**

**Part A. Logistic association models**

| **OSA definition** | **Label strategy** | **Adjustment** | **N (cases/controls)** | **Prevalence** | **OR per 0.1 unit (95% CI)** | **OR per SD (95% CI)** | **P** | **Status** |
| --- | --- | --- | --- | --- | --- | --- | --- | --- |
| pre_MHQ_expanded | full | age_sex | 620 (48/572) | 7.7% | 1.062 (1.031 to 1.095) | 1.779 (1.332 to 2.376) | <0.001 | ok |
| pre_MHQ_expanded | full | age_sex_bmi | 619 (48/571) | 7.8% | 1.034 (0.978 to 1.092) | 1.370 (0.810 to 2.319) | 0.241 | ok |
| pre_MHQ_expanded | strict | age_sex | 404 (48/356) | 11.9% | 1.084 (1.048 to 1.121) | 2.161 (1.564 to 2.986) | <0.001 | ok |
| pre_MHQ_expanded | strict | age_sex_bmi | 403 (48/355) | 11.9% | 1.042 (0.978 to 1.110) | 1.479 (0.810 to 2.700) | 0.203 | ok |
| ever_expanded | full | age_sex | 896 (59/837) | 6.6% | 1.063 (1.035 to 1.091) | 1.776 (1.387 to 2.274) | <0.001 | ok |
| ever_expanded | full | age_sex_bmi | 894 (59/835) | 6.6% | 1.026 (0.976 to 1.079) | 1.274 (0.794 to 2.044) | 0.316 | ok |
| ever_expanded | strict | age_sex | 598 (59/539) | 9.9% | 1.081 (1.050 to 1.113) | 2.068 (1.575 to 2.715) | <0.001 | ok |
| ever_expanded | strict | age_sex_bmi | 596 (59/537) | 9.9% | 1.031 (0.975 to 1.089) | 1.323 (0.788 to 2.222) | 0.290 | ok |
| pre_MHQ_ICD_defined | full | age_sex | Not estimable |  |  |  |  | Sparse or invalid covariate structure |
| pre_MHQ_ICD_defined | full | age_sex_bmi | Not estimable |  |  |  |  | Sparse or invalid covariate structure |
| pre_MHQ_ICD_defined | strict | age_sex | Not estimable |  |  |  |  | Sparse or invalid covariate structure |
| pre_MHQ_ICD_defined | strict | age_sex_bmi | Not estimable |  |  |  |  | Sparse or invalid covariate structure |
| ever_ICD_defined | full | age_sex | 330 (12/318) | 3.6% | 1.017 (0.947 to 1.092) | 1.135 (0.659 to 1.954) | 0.648 | ok |
| ever_ICD_defined | full | age_sex_bmi | 329 (12/317) | 3.6% | 0.958 (0.839 to 1.094) | 0.720 (0.261 to 1.989) | 0.527 | ok |
| ever_ICD_defined | strict | age_sex | 232 (12/220) | 5.2% | 1.023 (0.950 to 1.101) | 1.186 (0.675 to 2.084) | 0.553 | ok |
| ever_ICD_defined | strict | age_sex_bmi | 231 (12/219) | 5.2% | 0.959 (0.838 to 1.097) | 0.728 (0.261 to 2.026) | 0.543 | ok |

Note: Primary and sensitivity OSA definitions are shown separately. Non-estimable definitions are retained with their status.

**Part B. Sex interaction**

| **N** | **Cases** | **Controls** | **Interaction OR** | **Wald P** | **LRT P** |
| --- | --- | --- | --- | --- | --- |
| 619 | 48 | 571 | 0.845 | 0.588 | 0.586 |

Note: The interaction test assesses whether the TyG-WHtR association differs by sex.

**Part C. Nonlinearity test**

| **N** | **Linear AIC** | **Spline AIC** | **Nonlinear LRT chi-square** | **df** | **Nonlinear P** |
| --- | --- | --- | --- | --- | --- |
| 619 | 303.078 | 302.982 | 4.096 | 2 | 0.129 |

Note: Spline-versus-linear likelihood-ratio testing assesses evidence of departure from linearity.

**Supplementary Table S8. Primary depressive-symptom burden model development and sensitivity analyses**

**Part A. Full-spectrum primary model versus extreme-group sensitivity model**

| **Label strategy** | **N (cases/controls)** | **Prevalence** | **AUROC (95% CI)** | **PR-AUC** | **Brier** | **Calibration intercept** | **Calibration slope** | **Threshold** |
| --- | --- | --- | --- | --- | --- | --- | --- | --- |
| Full spectrum (primary) | 724 (63/661) | 8.7% | 0.726 (0.645 to 0.801) | 0.236 | 0.075 | -0.505 | 0.769 | 0.117 |
| Extreme-group sensitivity | 478 (63/415) | 13.2% | 0.739 (0.666 to 0.812) | 0.368 | 0.100 | -0.229 | 0.862 | 0.165 |

Note: The full-spectrum model is primary. The strict model excludes intermediate PHQ-2 scores and is treated only as a sensitivity analysis.

**Part B. Bootstrap uncertainty for primary nested-OOF performance**

| **Metric** | **Estimate** | **95% bootstrap CI** | **Successful bootstrap samples** |
| --- | --- | --- | --- |
| AUROC | 0.726 | 0.645 to 0.801 | 2,000 |
| PRAUC | 0.236 | 0.158 to 0.338 | 2,000 |
| Brier | 0.075 | 0.061 to 0.090 | 2,000 |
| sensitivity | 0.587 | 0.463 to 0.709 | 2,000 |
| specificity | 0.809 | 0.778 to 0.837 | 2,000 |
| false_positive_rate | 0.191 | 0.163 to 0.222 | 2,000 |
| calibration_intercept | -0.505 | -1.461 to 0.491 | 2,000 |
| calibration_slope | 0.769 | 0.378 to 1.242 | 2,000 |

Note: Bootstrap confidence intervals were calculated from pooled nested out-of-fold predictions.

**Part C. Non-zero coefficients of the locked primary model**

| **Term** | **Coefficient** |
| --- | --- |
| (Intercept) | -2.6347 |
| age | -0.4978 |
| bmi | 0.2186 |
| height_cm | -0.1465 |
| whtr | 0.0089 |
| glucose_mg_dl | 0.0486 |
| tyg_bmi | 0.0485 |
| daytime_dozing_X0 | -0.0365 |
| daytime_dozing_X2 | 0.4379 |
| self_rated_health_X2 | -0.2652 |
| self_rated_health_X4 | 0.6729 |
| walking_pace_X1 | 0.2282 |
| walking_pace_X3 | -0.1372 |

Note: Coefficients correspond to the final locked Version B elastic-net model. Zeroed coefficients are omitted from this compact table.

**Supplementary Table S9. Clinical evaluation of the locked depressive-symptom burden model in the complete 135-patient single-center clinical evaluation cohort**

**Part A. Cohort composition**

| **Clinical evaluation cohort** | **N** | **Female / male** | **HADS-D ≥8 cases / controls** | **Prevalence** |
| --- | --- | --- | --- | --- |
| Complete 135-patient single-center clinical evaluation cohort | 135 | 24 / 111 | 89 / 46 | 65.9% |

**Note:** The revised clinical evaluation is reported for the complete 135-patient cohort.

**Part B. Locked-model performance**

| **Endpoint** | **N (cases / controls)** | **AUROC (95% CI)** | **PR-AUC / prevalence** | **Brier** | **Sensitivity** | **Specificity** | **FPR** | **Calibration intercept** | **Calibration slope** |
| --- | --- | --- | --- | --- | --- | --- | --- | --- | --- |
| HADS-D ≥8 (primary clinical symptom-burden endpoint) | 135 (89 / 46) | 0.809 (0.738 to 0.879) | 0.907 / 0.659 | 0.356 | 0.843 | 0.500 | 0.500 | 2.556 | 1.181 |
| HADS-D ≥11 (sensitivity endpoint) | 135 (49 / 86) | 0.708 (0.614 to 0.802) | 0.624 / 0.363 | 0.216 | 0.837 | 0.337 | 0.663 | 0.458 | 0.771 |

**Note:** The model, preprocessing, and operating threshold were applied without refitting. PR-AUC is shown together with endpoint prevalence because precision-recall performance is prevalence dependent. HADS-D thresholds are used as symptom-burden categories rather than psychiatric diagnoses.

**Supplementary Table S10. Sex composition and exploratory sex-specific clinical evaluation**

**Part A. Sex composition**

| **Sex** | **n** | **Cases / controls** | **Prevalence** | **Mean age** | **Mean BMI** | **Mean TyG-WHtR** |
| --- | --- | --- | --- | --- | --- | --- |
| Female | 24 | 19 / 5 | 79.2% | 44.0 | 28.65 | 5.314 |
| Male | 111 | 70 / 41 | 63.1% | 40.3 | 27.89 | 5.229 |

**Part B. Sex-stratified discrimination and calibration**

| **Sex** | **N (cases/controls)** | **AUROC (95% CI)** | **PR-AUC** | **Brier** | **Sensitivity** | **Specificity** | **Calibration intercept** | **Calibration slope** | **Stability note** |
| --- | --- | --- | --- | --- | --- | --- | --- | --- | --- |
| Female | 24 (19/5) | 0.889 (0.731 to 1.000) | 0.970 | 0.413 | 0.789 | 0.800 | 5.139 | 1.788 | Exploratory: small sample or sparse outcome class |
| Male | 111 (70/41) | 0.805 (0.727 to 0.884) | 0.895 | 0.343 | 0.857 | 0.463 | 2.442 | 1.229 | More stable |

Note: Female estimates should be interpreted cautiously because the female subgroup contains few controls.

**Part C. Formal sex-related calibration tests**

| **Test** | **P value** |
| --- | --- |
| Sex-related calibration intercept shift | 0.028 |
| Sex-by-model-logit calibration slope interaction | 0.503 |

**Part D. Female-minus-male AUROC difference**

| **Contrast** | **AUROC difference** | **95% bootstrap CI** | **Successful bootstrap samples** |
| --- | --- | --- | --- |
| Female minus Male AUROC | 0.084 | -0.118 to 0.231 | 1,995 |

Note: The bootstrap confidence interval should be emphasized rather than the point estimate alone.

**Supplementary Table S11. Continuous HADS-D and depressive-symptom burden across model-based risk strata**

**Part A. Continuous HADS-D association**

| **N** | **HADS-D mean ± SD** | **HADS-D median** | **Spearman rho: model risk vs HADS-D** | **P value** |
| --- | --- | --- | --- | --- |
| 135 | 9.04 ± 3.09 | 9 | 0.456 | <0.001 |

Note: This analysis evaluates risk ranking against continuous symptom burden rather than treating HADS-D as a psychiatric diagnosis.

**Part B. HADS-D burden across predicted-risk quartiles**

| **Risk quartile** | **n** | **Mean predicted probability** | **HADS-D mean ± SD** | **HADS-D median** | **HADS-D ≥8** | **HADS-D ≥11** |
| --- | --- | --- | --- | --- | --- | --- |
| Q1 | 33 | 0.054 | 7.36 ± 2.66 | 7 | 36.4% | 18.2% |
| Q2 | 34 | 0.157 | 8.74 ± 3.29 | 8 | 55.9% | 32.4% |
| Q3 | 34 | 0.266 | 8.82 ± 2.94 | 9 | 70.6% | 29.4% |
| Q4 | 34 | 0.461 | 11.21 ± 2.16 | 12 | 100.0% | 64.7% |

Note: HADS-D thresholds are shown descriptively to characterize symptom burden across model-based risk strata.

**Supplementary Table S12. Original clinical evaluation and apparent post-hoc recalibration**

| **Model / update** | **N (cases/controls)** | **AUROC** | **PR-AUC** | **Brier** | **Calibration intercept** | **Calibration slope** | **Sensitivity** | **Specificity** | **FPR** | **PPV** | **NPV** | **Accuracy** | **F1** |
| --- | --- | --- | --- | --- | --- | --- | --- | --- | --- | --- | --- | --- | --- |
| Original locked model | 135 (89/46) | 0.809 | 0.907 | 0.356 | 2.556 | 1.181 | 0.843 | 0.500 | 0.500 | 0.765 | 0.622 | 0.726 | 0.802 |
| Post-hoc intercept-only update | 135 (89/46) | 0.809 | 0.907 | 0.173 | -0.100 | 1.181 | 1.000 | 0.000 | 1.000 | 0.659 |  | 0.659 | 0.795 |
| Post-hoc intercept+slope update | 135 (89/46) | 0.809 | 0.907 | 0.174 | 0.000 | 1.000 | 1.000 | 0.022 | 0.978 | 0.664 | 1.000 | 0.667 | 0.798 |

Note: Post-hoc recalibration was performed within the same 135-patient evaluation dataset. The updated calibration estimates are therefore apparent/local estimates and must not be presented as independent validation. Threshold-based operating characteristics after recalibration are descriptive because the original locked threshold was not re-optimized.

**Supplementary Table S13. Definitions, analytical roles, and sample sizes of the study populations**

| **Analytical population** | **Sample size** | **Female / male** | **Primary analytical role** | **Definition / source** | **Analytical interpretation** |
| --- | --- | --- | --- | --- | --- |
| Single-center deep-phenotyping cohort | 108 | 12 / 96 | Direct PSG, metabolic, and neuropsychological clinical association analyses | Single-center PSG-defined adult OSA | Used exclusively for single-center deep clinical phenotyping and cross-sectional clinical association analyses. |
| UKB general-population Olink analyses | Protein-specific N; median COG / PHQ-2 / GAD-2 = 14,412 / 13,630 / 13,612 | Outcome-level source cohorts: COG 9,072 / 7,742 PHQ-2 9,202 / 7,252 GAD-2 9,180 / 7,248 | Full-proteome population molecular-context analysis | Olink + same-wave outcome + required covariates; OSA not required | General-population molecular context; not an OSA-specific proteomic discovery cohort. |
| Available-source OSA TyG-WHtR association cohort | 619 | 137 / 482 | TyG-WHtR association with depressive-symptom burden | Pre-MHQ available-source OSA; complete cases for the primary adjusted association | UKB analytical subset distinct from the single-center PSG cohorts. |
| UKB depressive-symptom burden model-development cohort | 724 | 173 / 551 | Nested-OOF risk-ranking model development | Pre-MHQ available-source OSA; full-spectrum primary label | UKB development sample and PHQ-2 outcome definition are distinct from the single-center HADS-D clinical evaluation cohort. |
| Single-center clinical evaluation cohort | 135 | 24 / 111 | Clinical evaluation of the locked UKB-developed model | Single-center clinical OSA evaluation; HADS-D symptom-burden endpoint | Used as the complete 135-patient single-center cohort for clinical evaluation of the locked UKB-developed model. |

This recruitment window overlapped with and extended beyond that of the n = 108 deep-phenotyping cohort. Participant-ID cross-checking confirmed that 72 participants (8 women and 64 men) were included in both clinical datasets; therefore, participant-level independence between the two datasets was not assumed, and the n = 135 analysis is described as a single-center clinical evaluation rather than independent external validation.

**Note:** Study populations are reported according to their distinct analytical roles. OOF, out-of-fold; OSA, obstructive sleep apnea; PHQ-2, Patient Health Questionnaire-2; GAD-2, Generalized Anxiety Disorder-2; PSG, polysomnography; HADS-D, Hospital Anxiety and Depression Scale depression subscale.
